# Differences in dogs’ and wolves’ human-directed greeting behaviour: facial expressions, body language, and the problem of human biases

**DOI:** 10.1007/s10071-025-01978-7

**Published:** 2025-07-03

**Authors:** Svenja Capitain, Gwendolyn Wirobski, Çağla Önsal, Giulia Pedretti, Valeria Bevilacqua, Sarah Marshall-Pescini, Friederike Range

**Affiliations:** 1 Domestication Lab, Konrad Lorenz Institute of Ethology, University of Veterinary Medicine Vienna, Vienna, 1210 Austria; 2 Comparative Cognition Group, Institute of Biology, Faculty of Science, Université de Neuchâtel, Neuchâtel, 2000 Switzerland; 3 Department of Chemistry, Life Science and Environmental Sustainability, University of Parma, 43124 Parma, Italy; 4 Department of Biology, University of Padova, 35121 Padova, Italy

**Keywords:** Facial expressions, Canids, Dogs, Wolves, Dog-human interaction, Domestication

## Abstract

Dogs and wolves communicate effectively with humans, yet differences in their human-directed facial expressions and the role of relationship strength in shaping these behaviours remain poorly understood. This study explored the facial expressions of human-socialized wolves and dogs when greeting a bonded or familiar human through a fence. We hypothesised that differences would arise due to the domestication process, shaped further by the strength of their relationship. Additionally considering the bidirectionality integral to greeting interactions, we explored whether humans show different facial displays toward dogs versus wolves, expecting stronger differences in less bonded human partners due to unconscious biases. There was little overall difference between wolves’ and dogs’ facial expressions. However, wolves mainly displayed attentive, forward-directed ears, whereas dogs exhibited more ear positions associated with ambivalence or submission, such as rotated and downward-pushed ears. Dogs spent more time in proximity, gazing and tail wagging towards the human than wolves while both species showed more displacement behaviours (paw lift, whining, yawn) with bonded than familiar human partners. Interestingly, humans displayed more frequent, intense, and positive facial expressions toward dogs than wolves, suggesting implicit biases in human attitudes that were only partially influenced by familiarity. These results highlight the complexity of (studying) human-animal interactions. To what extent dogs’ submissive yet human-seeking behaviour is indeed species-specific, or rather results from biased human treatment during their life, and which specific mechanisms drove the likely bidirectional influence remains to be explored.

**Supplementary Information:**

The online version contains supplementary material available at 10.1007/s10071-025-01978-7.

## Introduction

Dogs (*Canis familiaris*) have shared their environment with humans more intimately than any other domesticate for more than 15,000 years (Larson and Fuller 2014; Larson et al. 2012). Throughout the domestication process, a multitude of selection pressures have shaped the initial wolfish ancestor into an animal that has come to reside inside human settlements around the globe (Hughes and Macdonald 2013). Considering our cooperative history of shared hunting and sheepherding, it had long been assumed that dogs were specifically selected for their improved communication skills with humans (Hare et al. 2010; Hare and Tomasello 2005). However, later comparison with extensively human-socialized wolves revealed that both wolves and dogs can communicate and cooperate successfully with humans, e.g. to retrieve food (Heberlein et al. 2016; Range et al. 2019). While this suggests that dogs’ communicative skills are not a novel adaptation, but rather, that they derive from the well-developed communicative capacities of their highly cooperative wolf ancestors (Range and Virányi 2015), this does not mean that their communication and signalling behaviour remained the same.

Behavioural differences that have received particular attention are the propensity to tail wag and gaze at the human, both of which are more frequently displayed by dogs than wolves (Bentosela et al. 2016; Burkhard et al. 2023; Gácsi et al. 2013). While the role of the tail wag is still unresolved (Leonetti et al. 2024), prolonged eye contact towards humans has been suggested to be an affiliative signal associated with oxytocin release in dog and in human partners (Nagasawa et al. 2015, (though see Kekecs et al. 2016 for commentary). Research into the genetic underpinnings of dogs’ changes in human-directed behaviour indicated that the selection pressures throughout domestication have led to an increased occurrence of structural variants in dogs’ genome that are associated with the William Beuren Syndrome in humans– a condition characterized by the exaggerated motivation to seek social contact (vonHoldt et al. 2017). Dogs’ frequently reported willingness to spend more time in human proximity and body contact compared to wolves (Bentosela et al. 2016; Lazzaroni et al. 2020; Wirobski et al. 2021) has therefore been interpreted in terms of hypersociability (Bentosela et al. 2016; vonHoldt et al. 2017).

Meanwhile, a different suite of communicative behaviours, in addition to hormonal evidence, has introduced an alternative explanation for dogs’ human proximity seeking (Wirobski et al. 2021, 2023). During human interactions, especially with a less familiar person, dogs showed more displacement behaviours and higher cortisol release than human-socialized wolves (Burkhard et al. 2023; Wirobski et al. 2021); both of which are frequently interpreted as signals of stress (Hekman et al. 2012), appeasement (Kuhne et al. 2014; Pedretti et al. 2023a), uncertainty (Pedretti, Canori, Marshall-Pescini, Pedretti et al. 2023a, b), negative arousal (Bremhorst et al. 2019), and/or submission (Mech and Boitani 2003; Schenkel 1967). This observation could be explained in terms of a selection throughout domestication for deferential behaviour, i.e. the willingness to yield to higher ranking individuals without challenge (Range et al. 2019). Here, the sustained human proximity may be understood as compliance with the wish of the human partner to interact, whereas wolves chose to only approach on their own terms (Lazzaroni et al. 2020; Wirobski et al. 2021).

Despite these advances and multiple hypotheses for dogs’ and wolves’ differences in human-directed behaviour, species comparisons have so far mainly focused on proximity, gazing, tail wagging, and displacement signals (Bentosela et al. 2016; Burkhard et al. 2023; Gácsi et al. 2013; Hansen Wheat et al. 2022; Lazzaroni et al. 2020; Wirobski et al. 2021). However, there is ample indication that dogs and wolves communicate through an intricate pattern of face and ear movements when interacting with conspecifics (Canori et al. 2025; Fox 1970; Maglieri et al. 2024; Mech 1999; Pedretti et al. 2024; Schenkel 1967). At least for dogs, it has also been evidenced that they show more facial expressions when a human is attending to them compared to being alone, suggesting interspecific communicative intent (Kaminski et al. 2017; Pedretti et al. 2022, 2024). How they differ from wolves’ facial expressions during human interactions and how domestication may have changed dogs’ interspecific facial communication has not yet been explored.

A valuable approach to understand facial behaviour is the Facial action coding system (FACS), an anatomically based method to objectively code and quantify facial muscle movements (initially developed for humans by Ekman and Friesen 1978; adapted for dogs by Waller et al. 2013). Therein, each visible movement is identified, categorized based on single or muscle group movements (“Action Units” (AUs), e.g. inner brow raiser, mouth movements) or “Action Descriptors” (ADs) that describe the movement of several muscles (e.g. jaw and ear movements). To the best of our knowledge, only two studies have used this system to compare dogs’ and wolves’ facial expressions to date. Kaminski et al. (2019) focussed on the inner eyebrow raiser, finding that it was more frequently raised in dogs compared to wolves, and that the muscle controlling it (levator anguli oculi medialis (LAOM), is largely absent in wolves. Hobkirk and Twiss (2024) used FACS to code the facial expressions of dogs and wolves in videos portraying different affective states. The subsequent linear discriminant analysis was able to successfully predict which affective state the video was associated with based on the facial expressions of the wolves. Interestingly, for the dogs, the distinctness of the facial expressions associated with each of the nine affective states was lower, with considerable confusion especially between friendly and fearful affective states (Hobkirk and Twiss 2024).

Understanding these differences in the human-directed context might provide an additional avenue for domestication research and beyond: Facial movements in animals, including humans, intentionally or unintentionally provide information about the sender’s emotional state and future action (Crivelli and Fridlund 2018; Waller et al. 2017). Systematic studies on what facial movements are associated with specific emotion-eliciting situations could hence present a promising approach to better understand internal motivations during different situations, including whether negative or positive affect prevailed during human contact. FACS have increasingly lent themselves to such research, revealing, for example, that forward-directed and adducted ears were associated with positive anticipation, attention, confidence, and dominance (Bremhorst et al. 2019, 2022; Caeiro et al. 2017; Fatjó et al. 2007; Pedretti et al. 2022), whereas ear flattening, ears downward, blinks, nose licks, and parted lips were more common in negative-arousal inducing situations (e.g. frustration, submission) (Boneh-Shitrit et al. 2022; Bremhorst et al. 2019, 2022; Fatjó et al. 2007; Pedretti et al. 2022).

To lay the groundwork for such associative investigations, explorations of the differences between facial movements in dogs and wolves in human-directed contexts are needed first. The present study hence set out to explore the spontaneous facial expressions of human-socialized, pack-living dogs and wolves in a 1-minute routine interaction– greeting through the fence– with known humans. To account for the role of familiarity and relationship strength that seemed central to the animals’ behaviour in previous studies, each animal interacted with a familiar and a bonded person separately.

Based on the previous findings on dogs’ sustained gazing, tail wagging, and proximity towards humans (Burkhard et al. 2023; Gácsi et al. 2013) and wolves’ clearer affective expressions in non-social situations (Hobkirk and Twiss 2024), we predicted differences in dogs’ and wolves’ facial expressions beyond the inner eyebrow raiser, further mediated by relationship strength (familiar vs. bonded). However, given the exploratory nature of this study, we did not make any predictions on which specific facial expressions might have been altered through the domestication process. Because previous studies found that dogs’ ear morphology (upright vs. floppy) affected some of their facial movements (Pedretti et al. 2022, 2024; Waller et al. 2013) and because all dogs in our study population had floppy ears, we additionally tested a cohort of pet dogs with upright ears (i.e. a similar ear conformation as wolves) in the same paradigm to check for the potential effect of morphology.

Inadvertently, constructing such an everyday interaction meant that we could blind the human partners to the hypotheses, but not to the species they interacted with. Given that societal attitudes and cultural stereotypes towards dogs and wolves diverge in the local cultures (Barmoen et al. 2024; Capitain et al. 2025; Fritts et al. 2003; Miklósi and Topál 2013; Serpell 2017), humans might hold explicit or unconscious (i.e. implicit) biases towards dogs and wolves (Jürgens and Hackett 2017). Such attitudes may become evident in subtle behaviours, body language, and facial expressions (Vanman et al. 2004). Moreover, one’s experience and relationship with the species might play a role in the perception of and behaviour towards them. While the people working with this study population are required to treat both species as similarly as possible, there is plenty of evidence that animals, including canines, react to subtle, often unconscious behaviours such as facial expressions, body odour, or tonality of vocalisations that humans might not be aware of themselves (Merola et al. 2014; Siniscalchi, d’Ingeo, Minunno, et al., 2018; Siniscalchi et al. 2018).

Hence, the second aim of this study was to explore whether humans’ facial expressions differed in a natural setting when interacting with a wolf vs. a dog. Because no other studies have explored this before, we only offer tentative predictions in consideration of the local cultural stereotypes and positive long-term experience (here sampled as relationship strength) with the animals. If the attitude towards dogs is indeed more positive than towards wolves, and/or dogs are perceived as safer interaction partners or more familiar, we would expect more positive, intense expressions in the dog condition compared to more negative or ambiguous expressions in the wolf condition. These differences in expression might be mediated by relationship strength with those dogs and wolves, which we would expect to result in smaller differences between the wolf and dog condition in humans who are more bonded with the animals.

## Materials & methods

### Ethical statement

The study received ethical approval from the ‘Ethik und Tierschutzkommission’ of the University of Veterinary Medicine Vienna (Reference Number: ETK-192/12/2022) and from the OPBA (Animal Welfare Committee) and REB (Research Ethics Board) of the University of Parma (Reference number:8-2023-N). The procedures adhere to the tenets of the Declaration of Helsinki. All human participants gave written informed consent for themselves and their dogs in case of the pet dogs.

### Subjects

The wolf-dog comparison included eleven wolves (8 males, 3 females, age 7 to 14 years) and eleven dogs (5 males, 6 females, age 1 to 9 years, all mongrels) housed at the Core Facility Wolf Science Center (CF-WSC) in Ernstbrunn, Austria. At the CF-WSC, all canids are hand-raised from an early age on and live in small conspecific groups with daily human contact. This handling regime ensures that wolves and dogs have similar experiences with humans throughout their lives, allowing for a fair comparison.

To control for the potential influence of ear morphology on facial movements of the wolves (erect ears) and CF-WSC dogs (floppy ears), an additional sample of ten pet dogs (5 males, 5 females, age 1 to 11 years, mixed shepherd breeds, all erect ears) were tested. Three pet dogs did not complete both test sessions, hence, the final sample size for the dog-dog comparison comprised eleven CF-WSC dogs and seven pet dogs.

The human partners were divided into a bonded and a familiar group. The ‘bonded’ group consisted of animal trainers (*n* = 4, age 31 to 46 years, all female) working at the CF-WSC. They all had hand-raised the respective animals they were tested with or had been working closely with them for at least half of the animal’s lifetime. The other group (‘familiar’) comprised researchers and students (*n* = 4, age 22 to 33 years, all female) who had been working at the CF-WSC for at least 6 months but without directly handling the animals. For the pet dogs, owners acted as the bonded person and a trainer from their dog training school acted as the familiar person.

### Greeting test

CF-WSC animals were tested individually in their home enclosures. The pet dogs were tested individually in a familiar, outdoor training area. Each animal was tested in a within-subject design with a bonded and a familiar person. The test consisted of two sessions on two separate days. In each session, the animal was greeted by both human partners in direct succession, with the order of bonded and familiar person counterbalanced between sessions and across dogs and wolves. The greeting test was set up to reflect a natural, daily greeting situation at the CF-WSC, whereby the human partner approached the animal in its enclosure separated by a fence.

**Fig. 1:**
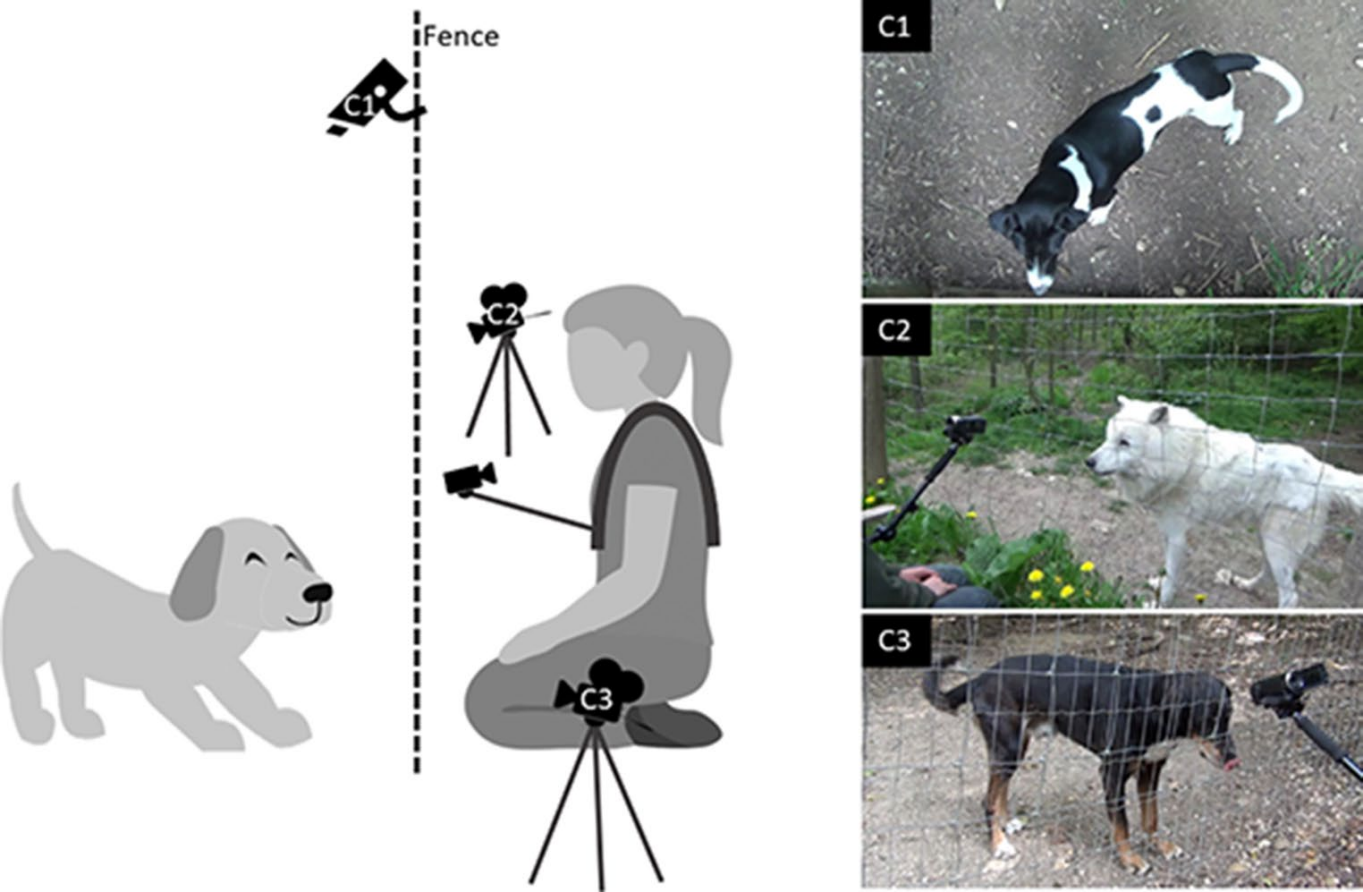
Test setup with the human and animal on either side of the fence and the perspectives of the three cameras (C1-3) angled at the animal. The fourth camera was angled at the person’s face with a chest harness

Hence, humans were instructed to act “as usual” and only received instructions where to crouch down and that they were not allowed to touch the animals. Before each session, the focal animal was shifted out of its home enclosure to an adjacent holding area (shifting compartment) by an assisting trainer not involved in the test, and three cameras were set up by the experimenter: two behind the fence facing the animal frontally from either side, and one fixed to the top of the fence filming the body and tail movements from above (Fig. 1). Out of sight of the animal, the first person was equipped with a chest harness that extended a camera stick to hold a camera approx. 50 cm in front of the person’s face to film their facial movements. To familiarize the animals with the harness, trainers had worn the chest harness for a week before the testing sessions took place.

At the start of the session, the focal animal was released back into the home enclosure by the assisting trainer to habituate to the recording equipment. Being shifted in and out of the enclosure and staying alone for more than ten minutes is a daily occurrence for the CF-WSC animals and was hence not expected to affect their disposition or behaviour. After two minutes, the first person walked up to the enclosure and crouched down right in front of the fence between the cameras. During the following minute, the person spontaneously talked and interacted with the animal as they would naturally, without moving towards the animal or touching it. After the minute had elapsed, she got up and walked out of sight of the focal animal. The chest harness was then handed to the second person during a 1-minute break, unseen by the animal. Then, the same procedure played out with the second person. The test was repeated on the second test day but with the order of the human partners reversed.

### Behavioural variables

The videos were coded with the open source software BORIS (Behavioural Observation Research Interactive Software) (v.8.25.4, Friard and Gamba 2016). For the animals, the facial expressions, including ear movements, were coded as defined by the Dog Facial Action Coding System (Dog FACS: Waller et al. 2013). To check whether our test setup reflecting a familiar, spontaneous interaction replicated previously reported behavioural differences between dogs and wolves, the duration and frequency of behaviours directed towards the human (e.g. proximity, tail wagging, gazing), vocalisations, and behaviours often interpreted as displacement (e.g. paw lifting, scratching, sniffing the environment (Beerda et al. 1997; Bremhorst et al. 2019; Pedretti et al. 2022)) were also coded. The ethogram with the exact behaviours and definitions can be found in Supplement Table 1. Only variables that occurred in more than 10% of all sessions were kept for the analysis (Table 1).

**Table 1:** Behavioural variables– dogs and wolves. Overview of the coded animal behaviours measured as duration (D) or frequency (F) that occurred in more than 10% of all sessions and were used for analysis. For facial and ear movements, the dog FACS indicators are presented in brackets. For detailed definitions see supplement table 1

Category	Behavioural variable	
Facial movements	• Inner brow raiser (AU101, D), • Blink (AU145, F) • Nose wrinkle and upper lip raiser (AU109 + AU110, D) • Upper lip raiser (AU110, D) • Lip corner puller (AU12, D) • Lip pucker (AU118, D)	• Lower lip depressor (AU116, D) • Yawning, mouth stretch (AU27, F) • Tongue show (AD19, D) • Lip wipe (AD37, F) • Nose lick (AD137, F) • Panting (AD126, D)
Ear movements	• Ears forward (EAD101, D) • Ears adductor (EAD102, D) • Ears flattener (EAD103, D)	• Ears rotator (EAD104, D), • Ears downward (EAD105, D)
Whole-body behaviours	• Tail wagging (D) • Head human-directed (gazing) (D) • Paw lift (D) • Head turn (F)	• Proximity < 1 body length (D) • Rubbing fence (D) • Sniffing environment (D)
Vocalisation	• Whining (D)	
Other	• Face not visible (D)	• Animal not visible (D)

Likewise, the Human FACS (Ekman et al. 2002; Ekman and Friesen 1978) was used to code the facial movements of the bonded and familiar person at the CF-WSC. The coder was blind to which species the human was interacting with. To decrease the complexity of this system, we included only the facial action units that were associated with positive or negative affect (Ekman et al. 2002), facial movements more frequently shown in the presence of dogs (e.g. inner and outer brow raiser, Gergely et al. 2023), or movements that appear in the Dog-FACS as well (e.g. mouth stretch, Waller et al. 2013) in previous studies (see Supplementary material Table 2). In addition, the five-level intensity scale suggested by the FACS manual (Ekman et al. 2002) was reduced to a two-level scale with levels A (“trace”), B (“slight”) and C (“marked”) collapsed into ‘low intensity’, and levels D (“severe”) and E (“extreme”) collapsed into ‘high intensity’. The only exception was AU12 (lip puller) that was separated into level B, C, and D + E to allow for appropriate definition of positive affect in line with the FACS manual (Ekman et al. 2002).

The facial movements and behaviours were all coded by FACS certified coders (VB, SC) who had undergone extensive training and reached acceptable reliability in the official Dog and Human FACS certification tests (Ekman et al. 2002; Waller et al. 2013) for their coded species respectively. 20% of the videos were re-coded for the whole-body and vocalisation behavioural ethogram that the coders did not undergo certification for. The interrater reliability was calculated as Intra-class correlation coefficients (ICCs (Rousson 2011), resulting in good interrater reliability (ICC > 0.80).

### Statistical analysis

To account for differences in the time animals spent near the cameras and visible on video, all behaviours (except time spent in proximity, which was analysed as a proportion of total test time) were analysed as their relative duration during proximity. The animals’ facial movements were analysed as relative durations (proportions) of the time their faces were visible while in proximity. Likewise, human facial movements were only analysed when the animal was within two body lengths of the human to ascertain that the facial movements corresponded to the greeting context. Because we were interested in the potential role of human behaviour biases overall, rather than the effect of individual facial movements, the coded human FACS were sorted into distinct categories of intensity ratio, expressivity, and valence for the analysis (Table 2).

**Table 2:** Behavioural variables– Human faces. Explanation of the categorization of behavioural variables for the coded human facial expressions. The exact definitions of the AUs can be found in supplement table 2

Category	Variable	Definition
Expressivity	Facial change (frequency):	The total frequency of shown facial movement (Sum of all AU frequencies)
	Facial range (frequency):	The total number of shown facial movement types (Sum of AU occurrence (binary yes/no per AU))
Intensity	Intensity Ratio	The ratio of the summed frequency of high and low intensity of all AUs
Valence	Positive valence (duration)	The summed duration of “true smiles” (AU12 intensity C or higher or AU12B + AU6, with no negative AUs present)
	Negative valence (duration)	The summed duration of facial expressions associated with negative emotions (Caeiro et al. 2017; Ekman et al. 2002): AU1 + 4, AU5, AU9, AU10, unilateral AU14, AU15, AU17, AU20

All statistical analyses were conducted in R (RStudio version 2023.06.0; Team 2023). To test the effect of species and relationship strength on the displayed behaviours, generalized linear mixed models (GLMMs) (Baayen et al. 2008) using the packages lme4 (Bates et al. 2015) and glmmTMB (Brooks et al. 2017) were fitted. The interaction term as well as the main effects of Species (CF-WSC wolf and CF-WSC dog) and Relationship strength (familiar and bonded) were included as test predictors. For the models using animal behaviours or facial movements as response variable, animal age (z-transformed), animal sex, and session number were included as control predictors (Head et al. 1997; Pedretti et al. 2024; Rosado et al. 2012). Animal ID, person ID, and dyad ID were included as random effects. For the models using the human facial variables as response variable, human age (z-transformed) and session were added as control predictors, and animal ID, person ID, and dyad ID were included as random effects. Random slopes were included where appropriate, with the respective factors manually dummy-coded and centred (see Statistical results, Supplementary material). Relative durations were analysed with a beta-distribution and frequencies with a Poisson distribution, with the time in proximity or face visible as an offset term. Models were examined for overdispersion, distribution of residuals, best linear unbiased prediction (BLUPs), and multicollinearity. Alternative distributions (negative binomial, zero-inflated, or binary) were employed wherever necessary (see Statistical results, Supplementary material). Furthermore, model stability was assessed by comparing the estimates obtained from the full model with those obtained from models with the levels of the random effects excluded one at a time. To keep the type I error rate at 5%, full-null model and reduced-null model comparisons were conducted using a likelihood ratio test (Dobson and Barnett 2018) with the null model lacking the test predictors, but comprising the control predictors and complete random effects structure. Significance of individual predictors in the final models as determined through single term deletions using the drop1 function are reported, and the model summaries of all factors are listed in the Supplementary material. Finally, Tukey-adjusted pairwise comparisons were employed using the emmeans package (Lenth 2023). Parametric bootstrapping was performed to obtain confidence intervals (function ‘boot.glmmtmb’ of glmmTMB).

For the CF-WSC and pet dog comparison, only the facial behaviours that differed between CF-WSC dogs and CF-WSC wolves were analysed to clarify whether this difference was due to species or rather morphological differences. To this end, the same model structure was used, with ‘CF-WSC dog’ and ‘pet dog’ as the two levels of the Species factor.

## Results

### Dog-wolf comparison

The interaction of the animals’ behaviour with relationship strength was only significant for two behaviours (rubbing the fence, whining). Hence, for all other models, we only report the additive effects of species and relationship strength here. For detailed statistical outcomes see the Statistical results in the Supplementary material.

**Fig. 2:**
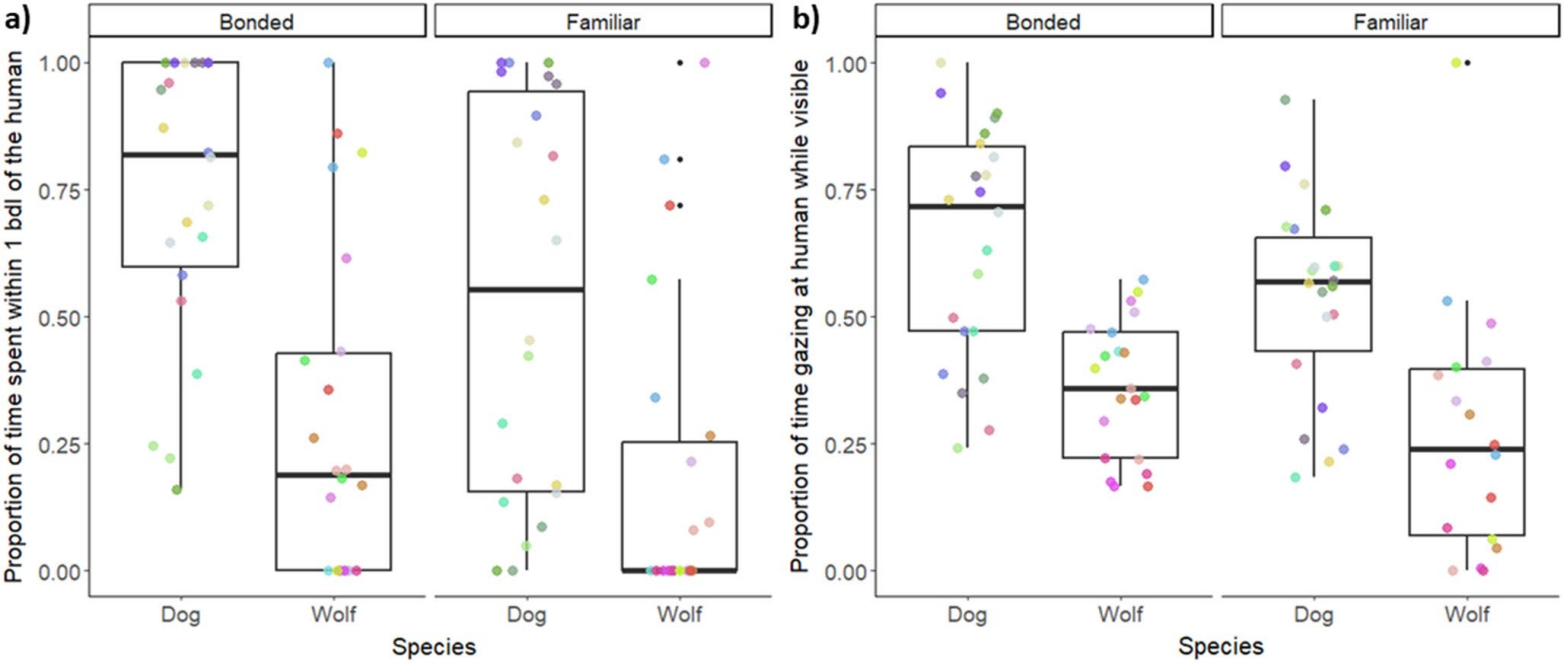
Dogs and wolves at the CF-WSC differed significantly in the proportion of time spent (**a**) within one body length (bdl) of the human and (**b**) gazing at the human. Both species spent more time close to the bonded than the familiar person (**a**). The colourful points depict the individual animals, with the same colour for each animal across Sessions and Relationship strength partners within each graph. Each box represents the interquartile range (25th to 75th percentile) of the behaviour, with the median marked by the thick line. Whiskers extend to the smallest and largest values within 1.5 times the interquartile range, and outliers are shown as black dots.

The time animals spent in proximity (within one body length) to the human partner was significantly influenced by species (χ² =14.44, df = 1, *p* < 0.001) and relationship strength (χ²=3.87, df = 1, *p* = 0.049). Dogs spent significantly more time in proximity with the human partners than wolves (est. =1.98 SE = 0.45, z = 4.36, *p* < 0.0001) and both species spent more time close to the bonded than the familiar partner (est.=0.58, SE = 0.26, z = 0.25, *p* = 0.024) (Fig. 2a). Moreover, dogs wagged their tail more (Species effect: χ²=24.7, df = 1, *p* < 0.001, post-hoc: est.=2.78, z.ratio = 5.87, *p* < 0.001) and gazed more at the human (regardless of relationship strength) than wolves (Fig. 2b) (Species effect: χ²=13.9, df = 1, *p* = 0.0002, post-hoc: est.=1.29, SE = 0.32, z.ratio = 4.08, *p* < 0.0001). Relationship strength affected paw lifting (χ²=6.75, df = 1, *p* = 0.009) with both species being more likely to show paw lifts towards the bonded than the familiar person (est.=1.76, SE = 0.76, z = 2.29, *p* = 0.02). The interaction of species and relationship strength significantly influenced the duration of rubbing against the fence (χ²=5.64, d = 1, *p* = 0.017) and marginally influenced the duration of whining (χ²=3.33, df = 1, *p* = 0.068), with dogs, but not wolves, rubbing longer (est.=1.22, SE = 0.27, z.ratio = 4.88, *p* < 0.0001) and whining longer (est.=1.36, SE = 0.36, z.ratio = 3.76, *p* = 0.001) when they were with a bonded rather than familiar person. Finally, head turning and the likelihood of sniffing the environment were not influenced by any of the predictors, and barking, scratching, and shaking were too infrequent to be analysed.

**Fig. 3:**
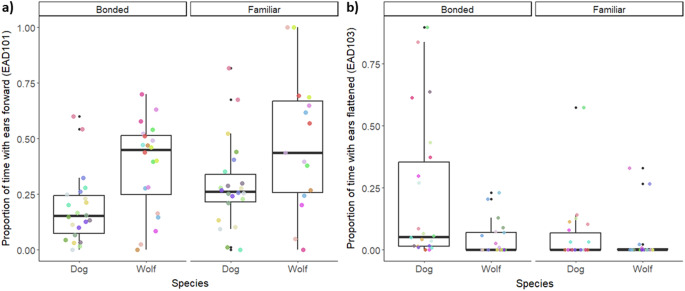
Dogs and wolves at the CF-WSC differed significantly in the proportion of time spent with (**a**) ears forward (EAD101), while (**b**) both species flattened their ears (EAD103) for longer during interactions with the bonded compared to the familiar human partner. The colourful points depict the individual animals, with the same colour for each animal across Sessions and Relationship strength partners within each graph. Each box represents the interquartile range (25th to 75th percentile) of the behaviour, with the median marked by the thick line. Whiskers extend to the smallest and largest values within 1.5 times the interquartile range, and outliers are shown as black dots.

In terms of facial movements, the inner brow raiser (AU101) differed between species (χ²=14.4, df = 1, *p* < 0.001). Specifically, dogs raised their inner brows for significantly longer than wolves (est.=1.52, SE = 0.37, z = 4.15, *p* < 0.001) (Fig. 3a). Dogs also raised their upper lip (AU110) for marginally longer than wolves (Species effect: χ²=3.9, df = 1, *p* = 0.048, post-hoc: est.=1.42, SE = 0.74, z = 1.9, *p* = 0.053). All other facial movements showed no significant difference between species. The lower lip depressor (AU116), lip pucker (AU118), lip corner puller (AU12), panting (AD126), blink (AD145), tongue show (AD19), lip wipe (AD37), and nose wrinkler in combination with lip raiser (AU109 + 110) were also not significantly influenced by relationship strength (Supplementary material). However, both species showed more mouth stretches (AU27, “yawn”) (Relationship strength effect: χ² = 7.5, df = 1, *p* = 0.006, post-hoc: est.= 1.81, SE = 0.77, z.ratio = 0.24, *p* = 0.018) and nose licks (AD137) (Relationship strength effect: χ² = 4.92, df = 1, *p* = 0.026, post-hoc: est.=0.63, SE = 0.27, z.ratio = 2.3, *p* = 0.02) while greeting the bonded compared to the familiar human partner.

Significant differences were also found in all ear movements except Ears adductor (EAD102). Dogs showed less ears forward (EAD101) than wolves (Species effect: χ²=4.3, df = 1, *p* = 0.038, post-hoc: est.=-0.83, SE = 0.36, z.ratio=-2.3, *p* = 0.022) (Fig. 3b), but rotated their ears more (EAD104) (Species effect: χ²=7.17, df = 1, *p* = 0.007, Post-hoc: est.=1.25, SE = 0.38, z.ratio = 3.3, *p* = 0.0009) and held their ears downwards (EAD105) for longer than wolves (Species effect: χ²=6.4, df = 1, *p* = 0.012, post-hoc: est.=0.93, SE = 0.31, z.ratio = 2.9, *p* = 0.003). Finally, there was an effect of relationship strength on ears forward (EAD101) (χ²=4.9, df = 1, *p* = 0.026) and ears flattener (EAD103) (χ²=3.9, df = 1, *p* = 0.048), with both species showing less ears forward (est.=-0.56, SE = 0.25, z.ratio=-2.25, *p* = 0.025) and more ears flattening (est.=0.49, SD = 0.24, z.ratio = 2.1, *p* = 0.037) towards the bonded compared to the familiar person.

**Fig. 4:**
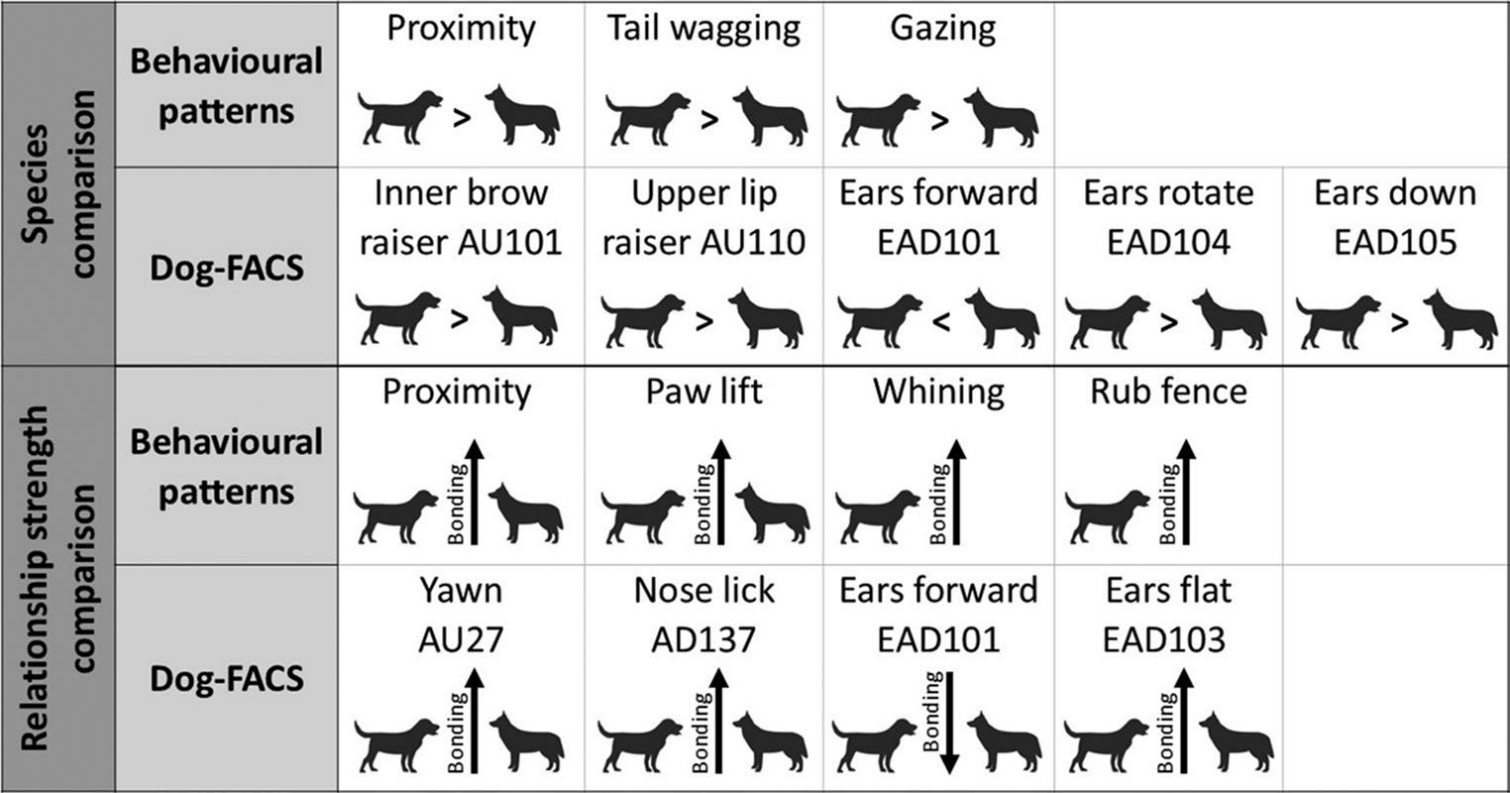
Summary of the differences between dogs and wolves and their behaviour towards a bonded or familiar person. Videos of the differing facial expressions in dogs and wolves can be found in Supplement Video 1

A summary of the differences between dogs and wolves is provided in Fig. 4 and in the Supplement Video 1.

### CF-WSC dog– pet dog comparison

The floppy-eared CF-WSC did not differ from the morphology control– the upright-eared pet dogs– in the facial movements (AU101 (χ²=0.09, df = 1, *p* = 0.76) and AU110 (χ²=1.0, df = 1, *p* = 0.11)) or the ear movements (EAD101 (χ²=2.51, df = 1, *p* = 0.11), EAD104 (χ²=1.3, df = 1, *p* = 0.25), EAD105 (χ²=2.27, df = 1, *p* = 0.13)) that had differed between the CF-WSC dogs and wolves.

### Comparison of human facial expressions while interacting with dogs or wolves

**Fig. 5:**
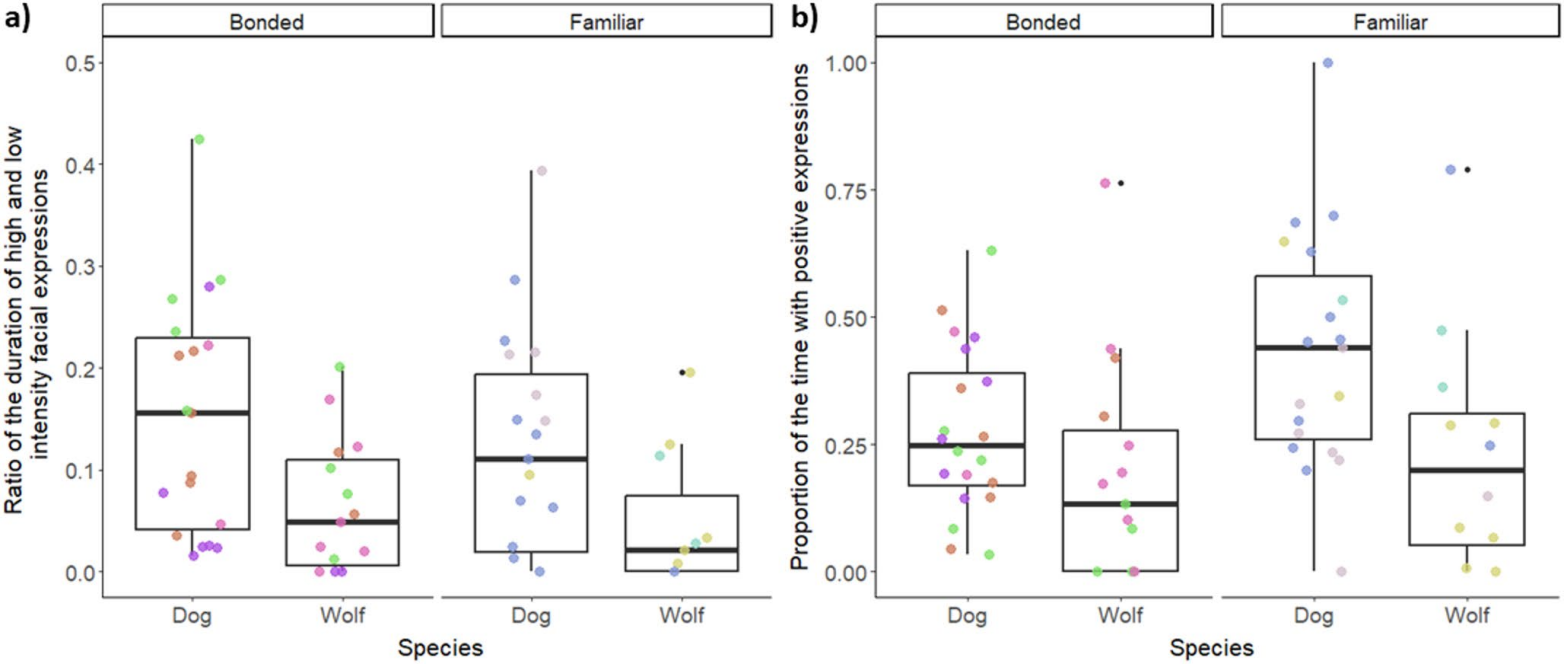
The human interaction partners showed (**a**) a significantly higher intensity in facial expressions when interacting with a dog compared to a wolf, and (**b**) expressed positive facial expressions for significantly longer in presence of a dog compared to a wolf. In the latter, there was a marginal interaction effect, but only the significant reduced model output is presented. The colourful points depict the individual human partners, with the same colour for each person across Sessions and animal partner within each graph. Each box represents the interquartile range (25th to 75th percentile) of the behaviour, with the median marked by the thick line. Whiskers extend to the smallest and largest values within 1.5 times the interquartile range, and outliers are shown as black dots.

Human partners showed a higher intensity of facial movements towards dogs than wolves regardless of relationship strength (Species effect: χ²=5.7, df = 1, *p* = 0.016, post-hoc (Dogs vs. wolves): est.=0.65, SE = 0.22, z.ratio = 3.0, *p* = 0.003) (Fig. 5a). The expressivity was also higher towards dogs than wolves, i.e. the human partners displayed more facial movements in total while greeting the CF-WSC dogs than wolves (“facial change”, Species effect: χ²=7.26, df = 1, *p* = 0.007, post-hoc (Dog vs. wolves): est.=0.449, SE = 0.16, z.ratio = 2.85, *p* = 0.004). Meanwhile, bonded human partners displayed a wider range of facial action units towards CF-WSC wolves than dogs, which was not the case in familiar interaction partners (Species x Relationship strength effect: χ²=3.73, df = 1, *p* = 0.054, post-hoc (Bonded dogs vs. Bonded wolves): est.=-1.32, SE = 0.22, z.ratio=-5.93, *p* < 0.001). Finally, the duration of negative facial expressions did not vary based on the species they interacted with (Species effect: χ²=0.01, df = 1, *p* = 0.91). However, the duration of positive facial expressions (“true smiles”) was marginally mediated by relationship strength (Species x Relationship strength effect: χ²=2.95, df = 1, *p* = 0.086). This was driven by the familiar partners showing significantly more positive facial expressions towards dogs compared to wolves (est.=1.31, SE = 0.47, z.ratio = 2.78, *p* = 0.028) and compared to the positive expressions the bonded persons showed towards dogs (est.=-1.12, SE = 0.367, z.ratio=-3.05, *p* = 0.012). Bonded partners did not differ in the duration of positive facial expressions they showed towards either species (est.=0.46, SE = 0.39, z.ratio = 1.13, *p* = 0.67) (Fig. 5b). Considering that the interaction effect was only a trend, further exploration of the reduced model revealed a species effect (χ²=3.55, df = 1, *p* = 0.043) with human partners showing positive facial expressions significantly longer towards dogs compared to wolves (Dogs vs. wolves: est.=0.75, SE = 0.35, z.ratio = 2.18, *p* = 0.029).

## Discussion

While both dogs and wolves can successfully cooperate and communicate with humans, past research suggests that domestication has led to differences in their human-directed behaviour (for an overview, see Range and Marshall-Pescini 2022). Using the Facial Action Coding System, we show here that dogs and wolves also differ in their facial expressions, particularly in their ear movements, during spontaneous, everyday greeting interactions with bonded and familiar humans through a fence (see Fig. 4 and Supplement Video 1 for an outcome overview). However, we also discovered that the humans diverged significantly in their facial expressions in interaction with dogs compared to wolves, which was only partially mediated by relationship strength and experience with these animals. We discuss the animals’ behavioural differences in terms of associated internal motivation and consider the implications the human results might have for this study and beyond.

Due to the differences we found in human facial expressions towards the animals, we start with a short but potentially integral detour to the human side before diving into the canine behaviours that this study initially aimed to investigate. While we had expected that the humans would somewhat differ in their behaviour towards dogs and wolves in light of the stereotypes associated with the species (Fritts et al. 2003; Jürgens and Hackett 2017; Serpell 2017) and the human participants’ experience with the animals across life, we were surprised to find species effects in all analysed categories– intensity, expressivity and valence– of the humans’ facial expressions, largely regardless of relationship strength. In more detail, both, the familiar and bonded human participants, showed more intense, more frequent, and more positive facial expressions towards dogs compared to wolves, despite the short interaction time in the experiment (1 min) and knowingly being observed. These results raise many questions, including where the differences might stem from and what they may mean in terms of how the people think about the animals, all of which we will tackle later in this discussion. More importantly in the context of the study aim, while it is possible that the human differences were a response to the animals’ species-intrinsic differences in human-directed behaviour, we cannot exclude the possibility that it was the humans’ behavioural differences that influenced the animals in the moment, or, perhaps, even across their lifetime. Given ample evidence that dogs and, though far less studied, wolves are somewhat influenced by (familiar) humans’ facial expression (Lazzaroni et al. 2023; Merola et al. 2014), subtle body language (Bräuer et al. 2024), intonation (Andics et al. 2016; Fonseca et al. 2023), and emotional chemosignals (Semin et al. 2019; Siniscalchi, d’Ingeo, Minunno, et al., 2018), the question is likely not *whether* the animals were influenced by the human, but rather, how strongly it influenced their behaviour. Consequently, we may question whether the differences we see between the species are indeed a result of domestication, or rather stem from different feedback experienced during their life with humans. Unfortunately, our study set up does not allow for the identification of temporal associations– who influenced whom, or which behaviours were particularly central. The following discussion on the animals’ behaviours hence cannot be interpreted in isolation from the human behaviour and our lack of understanding the cause-and-effect relationship.

Starting with the animals’ more general behaviour, our study mirrors recent evidence that dogs show more proximity seeking, gazing, and tail wagging towards humans than wolves (Bentosela et al. 2016; Lazzaroni et al. 2020; Wirobski et al. 2021). As expected, relationship strength played a considerable role for the animals’ greeting behaviour, with both species showing more nose licks, yawns, and paw lifts in the presence of the bonded compared to the familiar person. These results differ somewhat from previous studies at the CF-WSC, where such potential displacement signals were shown more often by dogs compared to wolves, in particular during body contact with a familiar rather than bonded person (Burkhard et al. 2023; Wirobski et al. 2021). Moreover, we found pronounced variation in the dogs’ willingness to stay close to the familiar person (Fig. 2a), a behaviour that had been characterized by a comparatively more homogenous, higher mean proportion of time spent with the familiar person in the previous study (Wirobski et al. 2021). Crucially, the human partners in our study were asked not to touch the animals, which might have lowered the need to appease, communicate or deal with stress on the dogs’ side (Hekman et al. 2012; Kuhne et al. 2014; Pedretti et al. 2023a). In isolation of the human behaviour, this interpretation, as well as the decrease in proximity, would be in line with the deferential behaviour hypothesis (Range et al. 2019) and previous research, positing that dogs might not stay in a humans’ proximity (purely) out of hypersocial motivation, but because they feel compelled to yield to the human’s wish for them to stay close (Lazzaroni et al. 2020; Wirobski et al. 2023). However, the question whether the diverging human (facial) behaviours might have been the cause of the species differences or whether these differences were an effect of domestication, and the humans reacted to the animals’ behaviours, remains an open question. Regardless, that dogs and wolves showed more signals and higher proximity towards the bonded person underlines not only that both species discriminate according to relationship strength, countering the gregariousness associated with hypersociability (Järvinen et al. 2013; vonHoldt et al. 2017), but also that socialized wolves actively seek human contact, although they remain in close proximity at a lower rate than dogs (Burkhard et al. 2023; Hansen Wheat et al. 2022; Wirobski et al. 2021). Given that there were no significant relationship strength effects on the human facial expressions (though they were marginal for the range and positive affect), these differences in the animals’ behaviour were more likely based on genuine bonding effects rather than responses to the human behaviour.

Subsequently, we focus on the so far almost unexplored differences in facial movements of dogs and wolves during a human greeting interaction. Contrary to our prediction, only two of the twelve facial expressions in the mouth, nose, and eye region differed between the species: more inner eyebrow raising and marginally more upper lip raising in dogs. Moreover, neither of these two has been associated with a specific internal state or the presence of an audience (suggesting intentional communication) in the literature so far. Initially, the inner eye brow raiser had been interpreted as an evolved communicative signal specific to dogs (Kaminski et al. 2017; Waller et al. 2013). However, Bremhorst et al. (2021) revealed a direct link of the inner eye brow raiser to eye movements rather than to the presence of an audience. Furthermore, the existence of the underlying muscle (LAOM) and the ‘puppy eyes expression’ has been demonstrated in coyotes (Cunningham et al. 2024) and African Wild Dogs (Felix et al. 2022), thus challenging the idea of evolution through human selection during domestication (Kaminski et al. 2019; Waller et al. 2013). Meanwhile, the upper lip raiser has been linked to jowl length (Caeiro et al. 2017) and reward dependency (Bremhorst et al. 2022), thus deeming it unsuitable for emotional description (Bremhorst et al. 2022). These results lend support to recent suggestions that dogs might be relying more on body rather than facial movements to communicate with us (Hobkirk and Twiss 2024; Siniscalchi, d’Ingeo, Minunno, et al., 2018). The selection for other morphological traits (Bradshaw et al. 2017; Hobkirk and Twiss 2024; Siniscalchi, d’Ingeo, Minunno, et al., 2018), humans’ difficulty to pick up on those subtle movements (Siniscalchi et al. 2018), or trouble with correct interpretation and focus on movements across species borders (Caeiro et al. 2017; Correia-Caeiro et al. 2020, 2021) have all been suggested as possible explanations. Whether there is indeed little difference between dogs and wolves in their visual communication with humans or whether they use their body differently to communicate with us remains to be explored.

Nevertheless, we did find considerable differences between the species’ ear movements during the interspecific greeting. Ear movements are prime communicative signals in canines, emerging as some of the most common and stable behaviours associated with affective states, often with communicative intent (Bremhorst et al. 2019, 2022; Pedretti et al. 2022; Siniscalchi, d’Ingeo, Minunno, et al., 2018). Forward-directed ear postures, which were more common in wolves compared to dogs in our study, have been associated with positive anticipation, attention, confidence, or dominance (Bremhorst et al. 2019, 2022; Fatjó et al. 2007; Pedretti et al. 2022; van der Borg et al. 2015). Ears downwards and ears rotator, on the other hand, more commonly displayed by the dogs than wolves in our sample, were linked to stress (Pedretti et al. 2022), frustration (Bremhorst et al. 2022) and uncertainty (Pedretti et al. 2024) in the literature. Furthermore, observational studies on canids’ social behaviour describe lowered and rotated ears as displays of submission and appeasement (Fox 1970; Mech 1999; Schenkel 1967; Spotte 2012) or ambiguity (‘airplane ears’ (Handelman 2012)). Taken together, these results suggest that the dogs may have been in a more submissive and perhaps conflicted state than wolves while greeting the human partners, while the wolves might have been in a more attentive state.

In contrast to previous studies (Pedretti et al. 2022, 2024), we did not find an influence of ear morphology (i.e. a difference between the floppy-eared CF-WSC dogs and the upright ear pet dogs) on facial expressions. Notably, these previous studies divided dogs in shepherd- and hunter-types, and they only focused on two affective states (frustration and anticipation) in an experimental setting (Pedretti et al. 2022, 2024). Meanwhile, a study on a wider emotional bandwidth in a more naturalistic context did not report these differences between breeds and ear morphology (Caeiro et al. 2017). It is hence possible that the effect of ear morphology is less evident during more natural conditions and/or when studying a wider range of emotions. Furthermore, the fact that the pet dogs did not differ from the CF-WSC dogs in these behaviours despite being tested in a familiar space rather than their home and having far less experience with the greeting through the fence suggests that the behavioural differences were more intrinsic to the greeting situation and the species rather than environmental factors.

One could now venture to interpret these behaviours in terms of species differences when greeting humans and even extend them to domestication questions– but again, in context of the lacking knowledge of cause-and-effect relationship with the human differences, these interpretations are hypothetical at this point, rather than conclusive, and highlight the need to tease these effects apart in the future. Intuitively, dogs’ more ambivalent affect could be interpreted as frustration with the fence, preventing them from satisfying their social motivation to get close to the human. Dogs’ higher propensity to rub the fence and whine when with the bonded human, which was not the case for wolves, might fall in line with this idea. However, the presence of the fence did not significantly alter the behaviour, including proximity and self-directed behaviours, of dogs with human handlers in a previous experiment (Wirobski et al. 2021). Moreover, the fact that this greeting is a daily, well-known interaction for these animals and that other facial movements repeatedly associated with frustration, namely blinking, ear flattening, and nose licking (Bremhorst et al. 2019, 2022; Pedretti et al. 2022), did not differ between species, somewhat undermines the support for this interpretation. Alternatively, or perhaps, additionally, one may return to the earlier interpretation of deferential behaviour, wherein dogs’ submissive ear postures towards both human partners, which were reserved for the bonded partner in case of the wolves, underlines their proposed inclination to view the human as dominant over them– regardless of relationship strength–, following the humans’ call despite feeling conflicted about the situation, while wolves only remain close when they are confident, attentive or interested, or to greet the dominant bonded partner (for similar suggestions see Lazzaroni et al. 2020; Wirobski et al. 2021).

On the other side of the fence, the exploration of human facial expressions revealed marked differences in the human partners’ behaviour towards the two species. We discuss two possible, not mutually exclusive explanations for these behavioural biases in the humans, with slightly different implications. One the one hand, the humans might have simply reacted to the differences in the species’ behaviour, for example by matching dogs’ intense gazing and tail wagging with more intense, friendly facial expressions, or toning down the intensity to honour wolves’ more reserved demeanour. Empathetic facial mirroring is a widely studied social facilitator in intraspecific human interactions (Lakin et al. 2003), and the effect of extended gazing on the oxytocin system (Nagasawa et al. 2015)– even if this was not replicated at the CF-WSC (Wirobski et al. 2021)– could lend further support for this theory. While it is still likely in this case that the response of the human to the animal might have created a feedback loop that confounds our species comparison, it might not have been causative to the species’ initial difference in approach behaviour.

The second explanation might be more complex. Human non-verbal behaviour, including facial expressions, can reflect conscious and unconscious biases towards interaction partners (Hess et al. 2022; McConnell and Leibold 2001; Weisbuch and Pauker 2011). In our canine context, earlier or more frequent exposure and cultural stereotypes are perhaps the most palpable origins of such biases (Fritts et al. 2003; Jürgens and Hackett 2017; Serpell 2017). Indeed, a study on human attitudes towards wild carnivores revealed an implicit negativity bias towards wolves regardless of whether or not the person feared them (Flykt et al. 2013). Given our evolutionary history with dogs, physiological mechanisms could also be at play, modulating positive, intense reactions when interacting with dogs rather than wolves (e.g. hormonal feedback loops (Nagasawa et al. 2015; but see Wirobski et al. 2021), attachment (Kurdek 2008; Kwong and Bartholomew 2011), repurposing infant directed behaviour with dogs (Gergely et al. 2023)). Moreover, while the people working at the CF-WSC are likely in favour of wolves and dogs, there are certain safety measures in place at the centre particularly in consideration of the wolves, which, although implemented mostly for the animals‘ sake, could potentially reinforce subconscious biases. For now, the causes remain at the level of speculation. However, counter to our expectations, the absence of effects of relationship strength on most of the variables suggests that these behavioural differences persist even in humans who enjoy working with these animals (Burkhard et al. 2023), who had long-term positive exposure with them, and who have endeavoured to treat them equally for years. Not only could this indicate that the human partners were not generally aware of these implicit biases, but also that the humans might have been showing subtle differences towards the two species throughout the animals’ lifetime.

Given the lacking knowledge about the underlying drivers on either side, it would be too far-fetched to speculate what the effect of the humans might have been on the animals’ behaviour we report in our study, particularly because the behavioural effects and mechanisms might differ between species (Fonseca et al. 2023). Future studies that elucidate the influence of one party on the other, e.g. using time series analyses and strictly controlling for the behaviour of one of the partners, will be integral next steps.

Despite all these considerations, we do, by no means, intend to blindly propagate that the humans’ behaviour was the sole cause of the differences between the species or that the discovery of human biases undermines the outcomes of past species comparisons with human involvement. There is evidence from past studies showing that dogs and wolves can disregard human preferences and biases, staying true to their own behavioural choices instead (Hegedüs et al. 2013; Topál et al. 2009). Rather, it is perceivable that it might have led to small shifts between studies and species, which might have contributed to the confusion between domestication hypotheses. Moreover, given that these human differences between species occurred unprompted, we might consider them as intrinsic to the human-canine relationship we aim to study. Perhaps, interspecific research with direct human interaction may thus have to contend with such lifetime variation for the sake of ecological validity. Using study methods to elicit the exact same behaviour of humans towards both species (e.g. video displays) could be a way to control for them within a study, but these might likewise have to gravel with the problem that the animals might be alienated by the unexpected human behaviour and the lack of immediate feedback. Considering the probable importance of such influences in future and past interpretations of different species’ human-directed behaviour far beyond our own, we urge for greater emphasis of this factor in interactive research on a biological, methodological and philosophical level.

Finally, we also need to emphasize that our sample size was limited and consisted of human participants that had extensive experience with both, wolves and dogs, compared to lay persons. Future studies should try to replicate our findings, and possibly use video or virtual reality experiences to increase human participant numbers and facilitate more control.

In conclusion, our results show that both dogs and wolves interacted with the human partner through proximity seeking, displacement behaviours, gazing, and differences in ear movements, with more proximity and submission towards the bonded compared to the familiar partner in both species. The relative lack of differences in facial movements between dogs and wolves compared to the extensive behavioural differences in their whole-body and ear movements could suggest that dogs rely more on the latter, more salient behaviours to communicate with us, though this speculation remains to be tested. While the ear movements suggested that wolves were more attentive and confident during the interaction, dogs’ averted ear positions point to a more ambiguous and submissive inner state. However, interpretations of this outcome in terms of intrinsic species differences are limited by biases in the human behaviour, with more intense, expressive, and positive facial expressions towards dogs compared to wolves, regardless of relationship strength. To our knowledge, this study is the first highlighting that humans implicitly display differences in their facial expressions towards dogs compared to wolves despite having received instructions to the contrary. However, given the nature of the study, it is unclear whether the animals influenced the humans or vice versa, and whether the influence extends to the species lifetime experience with humans. Considering that this influence, in turn, could affect our measurement and interpretation of the species’ behaviour towards us, and eventually also influence our thinking about domestication and formulation of hypotheses, further studies are clearly needed to pinpoint the mutual influence and causality of effects. Experimentally controlled studies that manipulate the facial expressions of one of the interaction partners and explore a broader range of affective states are necessary to understand the mutual influence, the underlying mechanisms of dogs’ seemingly deferential behaviour, and which behaviours may have evolved specifically for intentional communication with humans.

## Electronic supplementary material

Below is the link to the electronic supplementary material.

Supplementary Material 1

Supplementary Material 2

Supplementary Material 3

## Data Availability

All data on the coded behaviours that support the findings of this study are included within this paper and its Supplementary Information files.
