# Supplementary material for: Differences in dogs’ and wolves’ human-directed greeting behaviour: facial expressions, body language, and the problem of human biases
Source: Anim Cogn. 2025 Jul 3;28(1):54. doi: 10.1007/s10071-025-01978-7 (PMC12226620; doi:10.1007/s10071-025-01978-7)
Supplement: Supplementary file 2 — Supplementary Material 2 [file 10071_2025_1978_MOESM2_ESM.docx]

**SUPPLEMENTARY MATERIAL**

Publication title

Differences in dogs’ and wolves’ human-directed greeting behaviour: Facial Expressions, Body language, and the problem of human biases

Authors

Svenja Capitain^1*^ & Gwendolyn Wirobski^1,2*^, Çağla Önsal ^1^, Giulia Pedretti^3^, Valeria Bevilacqua^4^, Sarah Marshall-Pescini^1^ & Friederike Range^1^

^1^ Domestication Lab, Konrad Lorenz Institute of Ethology, University of Veterinary Medicine Vienna, 1210 Vienna, Austria

² Comparative Cognition Group, Institute of Biology, Faculty of Science, Université de Neuchâtel, 2000 Neuchâtel, Switzerland

³ Department of Chemistry, Life Science and Environmental Sustainability, University of Parma, 43124 Parma, Italy

^4^ Department of Biology, University of Padova, 35121 Padova, Italy

*Shared first authorship

Corresponding Author: [Svenja.capitain@vetmeduni.ac.at](mailto:Svenja.capitain@vetmeduni.ac.at), [Friederike.range@vetmeduni.ac.at](mailto:Friederike.range@vetmeduni.ac.at)

Journal

Animal Cognition

Content

[**Supplement Table 1: Dog and Wolf ethogram** 3](#_Toc195864537)

[**Supplement Table 2: Human ethogram** 5](#_Toc195864538)

[**Supplement Table 3: Statistical analysis** 7](#_Toc195864539)

[Statistical analysis 3.1: Dog-Wolf comparison 7](#_Toc195864540)

[**1)** **Proximity** 7](#_Toc195864541)

[**2)** **Tail wagging** 7](#_Toc195864542)

[**3)** **Gazing at human** 8](#_Toc195864543)

[**4)** **Paw lifting** 9](#_Toc195864544)

[**5)** **Rubbing along the fence** 9](#_Toc195864545)

[**6)** **Whining** 10](#_Toc195864546)

[**7)** **Head turn** 11](#_Toc195864547)

[**8)** **Sniffing the environment** 11](#_Toc195864548)

[**9)** **Too infrequent to be analysed** 11](#_Toc195864549)

[**10)** **Inner brow raiser (AU101)** 11](#_Toc195864550)

[**11)** **Upper lip raiser (AU110)** 12](#_Toc195864551)

[**12)** **Lower lip depressor (AU116)** 13](#_Toc195864552)

[**13)** **Lip pucker (AU118)** 13](#_Toc195864553)

[**14)** **Lip corner puller (AU12)** 13](#_Toc195864554)

[**15)** **Panting (AD126)** 13](#_Toc195864555)

[**16)** **Blink (AU145)** 13](#_Toc195864556)

[**17)** **Tongue show (AD19)** 14](#_Toc195864557)

[**18)** **Lip wipe (AD37)** 14](#_Toc195864558)

[**19)** **Nose wrinkle and upper lip raiser (AU109 + AU110)** 14](#_Toc195864559)

[**20)** **Yawn / Mouth stretch (AU27)** 14](#_Toc195864560)

[**21)** **Nose licks (AD137)** 15](#_Toc195864561)

[**22)** **Ears forward (EAD101)** 15](#_Toc195864562)

[**23)** **Ears Adductor (EAD102)** 16](#_Toc195864563)

[**24)** **Ears Flattener (EAD103)** 16](#_Toc195864564)

[**25)** **Ears Rotator (EAD104)** 17](#_Toc195864565)

[**26)** **Ears Downward (EAD105)** 17](#_Toc195864566)

[Statistical analysis 3.2: CF-WSC Dogs – Pet Dog comparison 19](#_Toc195864567)

[**1)** **Inner brow raiser (AU101)** 19](#_Toc195864568)

[**2)** **Upper lip raiser (AU102)** 19](#_Toc195864569)

[**3)** **Ears forward (EAD101)** 19](#_Toc195864570)

[**4)** **Ear Rotator (EAD104)** 20](#_Toc195864571)

[**5)** **Ears downward (EAD105)** 20](#_Toc195864572)

[Statistical analysis 3.3: Human facial reactions towards dogs vs. wolves 21](#_Toc195864573)

[**1)** **Expressiveness: Facial change (sum of all AU frequencies)** 21](#_Toc195864574)

[**2)** **Expressiveness: Facial range (sum of all occurred AUs)** 21](#_Toc195864575)

[**3)** **Intensity Ratio** 22](#_Toc195864576)

[**4)** **Valence: Duration of AUs associated with negative emotions (Ekman et al., 2002)** 23](#_Toc195864577)

[**5)** **Valence: Duration of AUs associated with positive emotions (Ekman et al., 2002)** 23](#_Toc195864578)

[**References** 24](#_Toc195864579)

# **Supplement Table 1: Dog and Wolf ethogram**

*Ethogram with the animal behaviours coded as duration (D) or frequency (F). Definitions of the facial movements followed Waller et al. (2013)’s Dog FACS descriptions. For the FACS with significant effects in the study, a video is provided in the supplement (****Supplement Video 1****).*

| **Category** | **Definition (FACS Code)** | **Description** | **Type of measure** |
| --- | --- | --- | --- |
| Ear movements | Ears forward (EAD101) | The ears are turned or pushed forward (rostrally). In dogs with pricked ears the pinna becomes rigid and tense. In dogs with floppy ears the base of the pinna is raised and pulled forward. Wrinkles may be formed along the midline frontal region. | D |
|  | Ears adductor (EAD102) | The ears are adducted, the base of the pinnas become closer together, by being pulled towards the head midline. The distance between the ears decreases. A vertical wrinkle may appear on the frontal region. | D |
|  | Ears flattener (EAD103) | Ears are pulled caudally, being flattened against the head. The pinna is pulled caudally, in the direction of the back of the head. In a frontal profile, the ears may disappear from the view. | D |
|  | Ears rotator (EAD104) | The ears are rotated laterally and externally. The internal part of the pinna is twisted out and outward-looking.  **Note**: The Dog FACS manual suggests to not code this for floppy-eared dogs, the movement has been observed in such dogs in other studies (Pedretti et al., 2024), hence they are included here | D |
|  | Ears downward (EAD105) | The ears are pulled ventrally, laterally. The base of the pinna in both ears moves away from each other. The distance between the ears increases. The frontal region skin is stretched, and the head takes a rounder shape. | D |
| Action Units | Inner brow raiser (AU101) | When the inner brow raises, it can be distinguished as a dorsal movement of the protuberance on the inner eye. The dog raises the soft protuberance present above the inner corner of the eye. Eyes become rounder and wider, and a vertical wrinkle may appear just between them. | D |
|  | Blink (AU145) | Upper and lower eyelids move towards together until they completely touch each other when the eyes close completely. In the AU145 eyes open again within half a second. | F |
|  | Nose wrinkle and upper lip raiser (AU109+ AU110) | In dogs the nose wrinkle (AU109) is only observed with AU110 (upper lip raiser) since they have only three muscles (levator nasolabialis, caninus, levator labii maxillaris) that work together for raising the upper lip and pulling the nose dorsally and/or caudally. These movements usually induce wrinkles formation on the muzzle and often come together with a snarl, causing the display of upper teeth. The upper lip raiser (AU110) can also be seen without the nose wrinkle (AU109), hence to the raise of the levator nasolabialis muscle alone. | D |
|  | Upper lip raiser (AU110) |  | D |
|  | Lip corner puller (AU12) | Due to the contraction of the zygomaticus muscle the lips' corners are pulled towards the ears, curving slightly. The mouth is opened, more teeth are visible, and some wrinkles may appear around the lips' corners as they're stretched. | D |
|  | Lip pucker (AU118) | The dog pushes the corners of its mouth forward (rostrally), the muzzle stays tense and some teeth may be visible if the mouths slightly opened. | D |
|  | Lower lip depressor (AU116) | Contrary to the neutral state with mouth open, in whom only the tips of the canines are visible, this movement makes other lower teeth, sometimes even the gums, visible as the lower lip's withdrawn ventrally. | D |
|  | Yawning, mouth stretch (AU27) | The mouth is stretch opened, and the lower jaw tensed (mutually exclusive of the AU26). Teeth are exposed, the tongue and the oral cavity are shown, and the lips are pulled back. | F |
| Action Descriptors | Tongue show (AD19) | The dog shows its tongue, the mouth is opened with the lower jaw dropped and lips parted | D |
|  | Lip wipe (AD37) | The tongue wipes the lips from the mouth midpoint till the mouth corner. | F |
|  | Nose lick (AD137) | The jaw is lowered, and the tongue licks the nose. It can be followed by a AD37 but they should be coded separately. Also flicks of the tongue are included. | F |
|  | Panting (AD126) | Tongue's shown, the lower jaw is dropped and there is no sign of tension. The dog breathes quickly and noisily through its mouth, while its chest moves rapidly. | D |
| Body behaviours | Head human-directed | The animal’s head is oriented towards the human. | D |
|  | Proximity <1 body length | The animal has at least one paw within one body length of the part of the fence that is closest to the human. | D |
|  | Wagging | The animal moves its tail back and forth. | D |
|  | Rubbing fence | Animal rubs its body or part of its body along the fence. | D |
|  | Paw lift | The animal stand or sit with one of its paw lifted from the ground without initiating locomotion. | D |
|  | Head turn | The animal is looking at the video and turns its head right or left. | F |
|  | Scratching | The animal is scratching its body with one of its paws. | D |
|  | Shaking | The animal shakes its body or its head from side to side. | F |
|  | Sniffing environment | Dog's muzzle gets closer to the floor or the fence, while its nostrils move quickly | D |
| Vocalisation | Whining | The animal is emitting high-pitched repeated vocalizations. | D |
|  | Barking | Loud and short typical vocalization, which may be repetitive. | D |
| Other | Face not visible | The face of the animal is not visible. | D |
|  | Animal not visible | The animal is not visible on camera. | D |

# **Supplement Table 2: Human ethogram**

*Ethogram with the coded human facial movements following and citing from the FACS manual by Ekman et al. (2002). Behaviours were coded as duration (D) or frequency (F).*

| **Area** | **Definitions (FACS Code)** | **Description** | **Type of measure** |
| --- | --- | --- | --- |
| Upper face | Inner brow raiser (AU1) | Pulls the inner portion of the eyebrows upwards | D |
|  | Outer brow raiser (AU2) | Pulls the lateral (outer) portion of the eyebrows upwards | D |
|  | Brow lowerer (AU4) | Lowers the eyebrow. It may be only the inner portion of the eyebrow that is lowered or it may be both inner and central portions that are lowered, or it may appear that the entire eyebrow is lowered. Pulls the eyebrows closer together | D |
|  | Upper lid raiser (AU5) | Widens the eye aperture. Raises the upper eyelid so that some or all of the upper eyelid disappears from view and more of the upper portion of the eyeball is exposed. | D |
|  | Cheek raiser (AU6) | Draws skin towards the eye from the temple and cheeks as the outer band of muscle around the eye constricts. Raises the infraorbital triangle, lifting the cheek upwards. Pushes the skin surrounding the eye towards the eye socket | D |
|  | Nose wrinkle (AU9) | Pulls the skin along the sides of the nose upwards towards the root of the nose causing wrinkles to appear along the  sides of the nose and across the root of the nose. | D |
| Lower face | Upper lip raiser (AU10) | Raises the upper lip. Center of upper lip is drawn straight up, the outer portions of upper lip are drawn up but not as  high as the center. | D |
|  | Lip corner puller (AU12) | Pulls the corners of the lips back and upward (obliquely) creating a U-shape to the mouth. Deepens the nasolabial furrow and raises the infraorbital triangle up. | D |
|  | Unilateral dimpler (AU14) | Tightens the corners of the mouth, pulling the corners somewhat inwards, and narrowing the lip corners. | D |
|  | Lip corner depressor (AU15) | Pulls the corners of the lips down which changes the shape of the lips so they are angled down at the corner, and usually the lower lip is somewhat stretched horizontally. | D |
|  | Chin raiser (AU17) | Pushes the chin boss and lower lip upward | D |
|  | Lip pucker (AU18) | Pushes the lips of the mouth forward and pulls medially, puckering the mouth. De-elongates the mouth opening, making the mouth opening smaller and rounder, and the lips appear tight and potentially protruding forward. | D |
|  | Lip stretcher (AU20) | Pulls the lips back laterally; the lip corners may be raised or lowered to a limited extent but the main movement is horizontal. Elongates the mouth. The lips become flattened and stretched by the lateral pull | D |
|  | Lip funneler (AU22) | Lips funnel outwards. Pulls in medially on the lip corners and exposes the teeth and may expose gums, often in the lower lip more than the upper. Exposes more of the red parts of the lips | D |
|  | Lip pressor (AU24) | Presses the lips together, without pushing up the chin boss. Narrowing and pressing of the center parts of the lips even though the lips themselves have not been pulled medially. | D |
|  | Lips part (AU25) | The lips part with parts of the insides of the mouth exposed | D |
|  | Mouth stretcher (AU27) | The mandible is pulled down. Mouth does not appear as if it has fallen open but as if it is actively pulled down forcibly or stretched open widely. | D |
|  | Jaw clencher (AU31) | A bulge appears far back along the jaw bone where it is hinged. The cheek adjacent to this bulge may become more  concave. | D |
|  | Lip bite (AU32) | You can see the teeth biting the lip (usually implying that another AU has lifted or lowered a lip so you can see the  teeth holding the other lip. | D |
|  | Lip wipe (AU37) | The tongue wipes the lips, moving back and forth or moving in just one direction. | F |
| Other | Speech (AU50) | The person is producing vocalisations. During vocalisations, other AUs involving lower face movements are not coded unless they persist beyond the lip movement that is needed for the creation of speech itself. |  |
|  | Not visible | 70 - brows and forehead, 71 - eyes, 72 - lower face, 73 - entire face, 74 - unscorable |  |

# **Supplement Table 3: Statistical analysis**

## Statistical analysis 3.1: Dog-Wolf comparison

Displayed are the full model, the full-null model comparison (using a likelihood ratio test (Dobson & Barnett, 2018)), the model summary with 95% confidence intervals (Parametric bootstrapping (function ‘boot.glmmtmb’ of glmmTMB)), and, where applicable, the post-hoc pair-wise comparison (emmeans package (Lenth, 2023)) for each model. If the full model with the higher order interaction (Species*Relationship strength) did not reveal significance, reduced models with the main effects as additive terms (Species + Relationship strength) were fitted, with all other factors remaining the same.

### **Proximity**

Full model: full.1bdl=glmmTMB(1body_PropDurT ~ Species*Relationship strength + Age.z + Sex +

Session + (1|AnimalID)+ (1+ Age.z + Sex.W||PersonID) + (1|Dyad),

family=beta_family, data=Cdata)

Full-null model comparison: χ²=18.511, DF=3, *p=*0.0003

Full model summary:

| Variable | Estimate | Std. Error | z-value | p-value |
| --- | --- | --- | --- | --- |
| SpeciesWolf:Relationship strengthFH | 0.2675 | 0.5035 | 0.531 | 0.5952 |

Reduced-full model comparison: χ²=18.23, DF=2, *p=*0.0001

Reduced model summary:

| Variable | Estimate | Std. Error | z-value | p-value | Sign. | Lower CI | Upper CI |
| --- | --- | --- | --- | --- | --- | --- | --- |
| (Intercept) | 1.212 | 0.370 | 3.272 | 0.0011 | ** | 0.464 | 1.979 |
| **Species**Wolf | -1.978 | 0.454 | -4.360 | <0.001 | *** | -2.948 | -1.100 |
| **Relationship strength**FH | -0.579 | 0.257 | -2.251 | 0.0244 | * | -1.124 | -0.093 |
| Age.z | 0.269 | 0.219 | 1.229 | 0.219 |  | -0.152 | 0.722 |
| SexW | -0.224 | 0.331 | -0.679 | 0.497 |  | -0.940 | 0.439 |
| Sessiontrial2 | -0.136 | 0.252 | -0.539 | 0.590 |  | -0.623 | 0.384 |

Pairwise comparison:

| contrast | Estimate | Std. error | Df | z-ratio | p-value |
| --- | --- | --- | --- | --- | --- |
| Dog - Wolf | 1.98 | 0.454 | Inf | 4.360 | <.0001 |
| Bonded – Familiar | 0.579 | 0.257 | Inf | 2.251 | 0.0244 |

### **Tail wagging**

Full model: full.wag=glmmTMB(WAG_PropDurV ~ Species*Relationship strength + Age.z + Sex +

Session + (1|AnimalID)+ (1+ Age.z + Sex.W||PersonID)+ (1|Dyad),

family=beta_family, data=Avdata)

Full-null model comparison: χ²=28.218, Df=3, p<0.0001

Full model summary:

| Variable | Estimate | Std. Error | z-value | p-value |
| --- | --- | --- | --- | --- |
| SpeciesWolf:Relationship strengthFH | 0.563712 | 0.440466 | 1.280 | 0.2006 |

Reduced-null model comparison: χ²=26.602, Df=2, p<0.0001

Reduced model summary:

|  | Estimate | Std. Error | z-value | p-value |  | Lower CI | Upper CI |
| --- | --- | --- | --- | --- | --- | --- | --- |
| (Intercept) | 0.339 | 0.331 | 1.025 | 0.305 |  | -0.335 | 1.058 |
| **SpeciesWolf** | -2.781 | 0.474 | -5.867 | <0.001 | *** | -3.806 | -1.901 |
| Relationship strengthFH | -0.328 | 0.221 | -1.481 | 0.139 |  | -0.787 | 0.150 |
| Age.z | -0.115 | 0.217 | -0.529 | 0.597 |  | -0.549 | 0.320 |
| SexW | 0.375 | 0.311 | 1.203 | 0.229 |  | -0.284 | 0.979 |
| Sessiontrial2 | 0.001 | 0.217 | 0.004 | 0.997 |  | -0.443 | 0.444 |

Pairwise comparison:

| contrast | Estimate | Std. error | Df | z-ratio | p-value |
| --- | --- | --- | --- | --- | --- |
| Dog - Wolf | 2.78 | 0.474 | Inf | 5.867 | <.0001 |

### **Gazing at human**

Full model: full.hhum=glmmTMB(Head_HUMAN_PropDurV ~ Species*Relationship strength +

Age.z + Sex + Session + (1|AnimalID) + (1+ Age.z + Sex.W||PersonID) + (1|Dyad),
 family=beta_family, data=Avdata)

Full-null model comparison: χ²=14.48, Df=3, *p=*0.0023

| Variable | Estimate | Std. Error | z-value | p-value |
| --- | --- | --- | --- | --- |
| SpeciesWolf:Relationship strengthFH | -0.01471 | 0.43715 | -0.034 | 0.973150 |

Full model summary:

Reduced-null model comparison: χ²=14.48, Df=2, *p=*0.0007

Reduced model summary:

|  | Estimate | Std. Error | z-value | p-value |  | Lower CI | Upper CI |
| --- | --- | --- | --- | --- | --- | --- | --- |
| (Intercept) | 1.128 | 0.330 | 3.413 | 0.001 | ** | 0.502 | 1.789 |
| **SpeciesWolf** | -1.287 | 0.316 | -4.080 | <0.001 | *** | -1.915 | -0.645 |
| Relationship strengthFH | -0.357 | 0.377 | -0.947 | 0.344 |  | -1.118 | 0.384 |
| Age.z | -0.078 | 0.176 | -0.442 | 0.659 |  | -0.424 | 0.276 |
| SexW | -0.066 | 0.208 | -0.318 | 0.750 |  | -0.463 | 0.340 |
| **Sessiontrial2** | -0.858 | 0.173 | -4.956 | <0.001 | *** | -1.230 | -0.500 |

|  |  |  |  |  |  |
| --- | --- | --- | --- | --- | --- |

Pairwise comparison:

| contrast | Estimate | Std. error | Df | z-ratio | p-value |
| --- | --- | --- | --- | --- | --- |
| Dog - Wolf | 1.29 | 0.316 | Inf | 4.080 | <.0001 |
| trial1 - trial2 | 0.858 | 0.173 | Inf | 4.956 | <.0001 |

### **Paw lifting**

Full model: full.paw.binary <- glmmTMB(Pawlift_BINARY ~ Species*Relationship strength + Age.z

+ Sex + Session + (1|AnimalID) + (1 PersonID) + (1|Dyad), family=binomial,

data=Avdata)

Full-null model comparison: χ²=22.33, Df=3, p<0.001

Full model summary:

| Variable | Estimate | Std. Error | z-value | p-value |
| --- | --- | --- | --- | --- |
| SpeciesWolf:Relationship strengthFH | 26.1605 | 17292.2377 | 0.002 | 0.9988 |

Reduced-null model comparison: χ²=9.18, Df=2, *p=*0.01

Reduced model summary:

|  | Estimate | Std. Error | z-value | p-value |  | Lower CI | Upper CI |
| --- | --- | --- | --- | --- | --- | --- | --- |
| (Intercept) | -0.333 | 0.787 | -0.423 | 0.672 |  | -3.110 | 1.536 |
| **Relationship strengthFH** | -1.761 | 0.766 | -2.299 | 0.022 | * | -5.335 | -0.557 |
| SpeciesWolf | -1.845 | 1.104 | -1.672 | 0.095 | . | -29.668 | 0.567 |
| Age.z | -0.765 | 0.487 | -1.570 | 0.116 |  | -3.647 | 0.128 |
| SexW | -0.478 | 0.767 | -0.623 | 0.533 |  | -2.643 | 1.236 |
| Sessiontrial2 | 0.723 | 0.666 | 1.085 | 0.278 |  | -0.760 | 2.909 |

Pairwise comparison:

| contrast | estimate | SE | df | z.ratio | p-value |
| --- | --- | --- | --- | --- | --- |
| Dog – Wolf | 1.84 | 1.1 | Inf | 1.672 | 0.0946 |
| Bonded – Familiar | 1.76 | 0.766 | Inf | 2.299 | 0.0215 |

### **Rubbing along the fence**

Full model: full.rub=glmmTMB(RUBBING_FENCE_PropDurV ~ Species*Relationship strength +

Age.z + Sex + Session + (1|AnimalID)+ (1+ Age.z + Sex.W||PersonID)+ (1|Dyad),

family=beta_family, data=Avdata)

Full-null model comparison: χ²=12.39, Df=3, *p=*0.006

Full model summary:

|  | Estimate | Std. Error | z-value | p-value |  | Lower CI | Upper CI |
| --- | --- | --- | --- | --- | --- | --- | --- |
| (Intercept) | -3.408 | 0.307 | -11.104 | <0.001 | *** | -4.200 | -3.004 |
| **Species**Wolf | -0.654 | 0.328 | -1.997 | 0.046 | * | -0.886 | 0.352 |
| **Relationship strength**FH | -1.228 | 0.274 | -4.488 | 0.000 | *** | -1.307 | -0.324 |
| Age.z | -0.278 | 0.149 | -1.869 | 0.062 | . | -0.639 | 0.050 |
| SexW | 0.115 | 0.470 | 0.244 | 0.807 |  | -0.769 | 1.053 |
| Sessiontrial2 | 0.155 | 0.152 | 1.018 | 0.309 |  | -0.158 | 0.452 |
| SpeciesWolf:Relationship strengthFH | 0.909 | 0.352 | 2.583 | 0.010 | ** | 3.953 | 4.831 |

Pairwise comparison:

| contrast | estimate | SE | df | z.ratio | p-value |
| --- | --- | --- | --- | --- | --- |
| Dog Bonded - Wolf Bonded | 0.654 | 0.328 | Inf | 1.997 | 0.1891 |
| **Dog Bonded - Dog Familiar** | 1.228 | 0.274 | Inf | 4.488 | <.0001 |
| Dog Bonded - Wolf Familiar | 0.973 | 0.368 | Inf | 2.642 | 0.0411 |
| Wolf Bonded - Dog Familiar | 0.573 | 0.381 | Inf | 1.503 | 0.4355 |
| Wolf Bonded - Wolf Familiar | 0.318 | 0.317 | Inf | 1.003 | 0.7475 |
| Dog Familiar - Wolf Familiar | -0.255 | 0.349 | Inf | -0.729 | 0.8853 |

### **Whining**

Full model: full.whine=glmmTMB(WHINE_PropDurV ~ Species*Relationship strength + Age.z +

Sex + Session + (1|AnimalID)+ (1+ Age.z + Sex.W||PersonID)+ (1|Dyad), family=beta_family, data=Avdata)

Full-null model comparison: χ²=8.98, Df=3, *p=*0.0295

Full model summary:

|  | Estimate | Std. Error | z-value | p-value |  | Lower CI | Upper CI |
| --- | --- | --- | --- | --- | --- | --- | --- |
| (Intercept) | -2.139 | 0.325 | -6.588 | <0.001 | *** | -2.868 | -1.525 |
| **Species**Wolf | -0.479 | 0.424 | -1.131 | 0.258 |  | -1.331 | 0.379 |
| **Relationship strength**FH | -1.360 | 0.362 | -3.761 | <0.001 | *** | -2.101 | -0.699 |
| Age.z | -0.889 | 0.205 | -4.333 | <0.001 | *** | -1.295 | -0.512 |
| SexW | -0.384 | 0.253 | -1.518 | 0.129 |  | -0.941 | 0.160 |
| Sessiontrial2 | -0.052 | 0.176 | -0.294 | 0.769 |  | -0.397 | 0.300 |
| SpeciesWolf:Relationship strengthFH | 1.097 | 0.467 | 2.351 | 0.019 | * | 0.094 | 2.143 |

Pairwise comparison:

| contrast | estimate | SE | df | z.ratio | p-value |
| --- | --- | --- | --- | --- | --- |
| Dog Bonded - Wolf Bonded | 0.479 | 0.424 | Inf | 1.131 | 0.671 |
| Dog Bonded - Dog Familiar | 1.360 | 0.362 | Inf | 3.761 | 0.001 |
| Dog Bonded - Wolf Familiar | 0.741 | 0.506 | Inf | 1.467 | 0.458 |
| Wolf Bonded - Dog Familiar | 0.881 | 0.478 | Inf | 1.841 | 0.254 |
| Wolf Bonded - Wolf Familiar | 0.262 | 0.414 | Inf | 0.634 | 0.921 |
| Dog Familiar - Wolf Familiar | -0.618 | 0.470 | Inf | -1.316 | 0.553 |

### **Head turn**

Full model: full.hturn=glmmTMB(HEADTURN_Freq ~ Species*Relationship strength + Age.z + Sex

+ Session + offset(AnimalVisible_Dur.z)+ (1|AnimalID)+

(1+ Sex.W + Age.z ||PersonID)+ (1|Dyad),

family=nbinom2, data=Avdata)

Full-null model comparison: χ²=6.71, Df=5, *p=*0.24

Reduced-null model comparison: χ²=2.06, Df=2, *p=*0.36

### **Sniffing the environment**

Full model: full.sniff=glmmTMB(SNIFFING_ENVIRONMENT_PropDurV ~ Species*Relationship
 strength + Age.z + Sex + Session + (1|AnimalID)+ (1+ Age.z + Sex.W||PersonID)+
 (1|Dyad), family=binomial, data=Avdata)

Full-null model comparison: χ²=4.42, Df=3, *p=*0.22

Reduced-null model comparison: χ²=2.99, Df=2, *p=*0.22

### **Too infrequent to be analysed**

**= occurred in <10% of interactions**

- Barking
- Scratching
- Shaking

### **Inner brow raiser (AU101)**

Full model: full.AU101=glmmTMB(AU101_PropDurFvv ~ Species*Relationship strength + Age.z +

Sex + Session + (1|AnimalID)+ (1+ Age.z + Sex.W||PersonID)+ (1|Dyad),

family=beta_family, data=Fvdata)

Full-null model comparison: χ²=16.181, Df=3, *p=*0.001

Full model summary:

| Variable | Estimate | Std. Error | z-value | p-value |
| --- | --- | --- | --- | --- |
| SpeciesWolf:Relationship strengthFH | 0.2417 | 0.3750 | 0.645 | 0.519 |

Reduced-null model comparison: χ²=15.77, Df=2, *p=*0.0004

Reduced model summary:

|  | Estimate | Std. Error | z-value | p-value |  | Lower CI | Upper CI |
| --- | --- | --- | --- | --- | --- | --- | --- |
| (Intercept) | -2.140 | 0.264 | -8.109 | <0.001 | *** | -2.721 | -1.652 |
| **Species**Wolf | -1.522 | 0.367 | -4.146 | <0.001 | *** | -2.279 | -0.871 |
| Relationship strengthFH | -0.241 | 0.179 | -1.349 | 0.177 |  | -0.604 | 0.110 |
| Age.z | 0.263 | 0.174 | 1.515 | 0.130 |  | -0.093 | 0.611 |
| SexW | -0.125 | 0.260 | -0.479 | 0.632 |  | -0.662 | 0.384 |
| Sessiontrial2 | -0.246 | 0.184 | -1.337 | 0.181 |  | -0.628 | 0.141 |

Pairwise comparison:

| contrast | estimate | SE | df | z.ratio | p-value |
| --- | --- | --- | --- | --- | --- |
| Dog – Wolf | 1.52 | 0.367 | Inf | 4.146 | <.0001 |

### **Upper lip raiser (AU110)**

Full model: full.AU110.binary <- glmmTMB(AU110_BINARY ~ Species*Relationship strength +

Age.z + Sex + Session + (1|AnimalID)+ (1+ Age.z + Sex.W||PersonID)+ (1|Dyad),

family=binomial, data=Fvdata)

Full-null model comparison: χ²=6.62, Df=3, *p=*0.08

Full model summary:

| Variable | Estimate | Std. Error | z-value | p-value |
| --- | --- | --- | --- | --- |
| SpeciesWolf:Relationship strengthFH | -0.58140 | -1.17130 | -0.496 | 0.6196 |

Reduced-null model comparison: χ²=6.38, Df=2, *p=*0.041

Reduced model summary:

|  | Estimate | Std. Error | z-value | p-value |  | Lower CI | Upper CI |
| --- | --- | --- | --- | --- | --- | --- | --- |
| (Intercept) | 0.943 | 0.623 | 1.513 | 0.130 |  | -0.298 | 2.735 |
| **Relationship strength**FH | -0.967 | 0.540 | -1.791 | 0.073 | . | -2.856 | 0.003 |
| **Species**Wolf | -1.424 | 0.736 | -1.934 | 0.053 | . | -3.859 | 0.056 |
| Age.z | 0.084 | 0.366 | 0.228 | 0.819 |  | -0.755 | 0.986 |
| SexW | 0.133 | 0.537 | 0.248 | 0.804 |  | -1.211 | 1.415 |
| Sessiontrial2 | -0.970 | 0.527 | -1.840 | 0.066 | . | -2.404 | -0.069 |

Pairwise comparison:

| contrast | estimate | SE | df | z.ratio | p-value |
| --- | --- | --- | --- | --- | --- |
| Dog – Wolf | 1.42 | 0.736 | Inf | 1.934 | 0.0531 |
| Bonded – Familiar | 0.967 | 0.54 | Inf | 1.791 | 0.0734 |

### **Lower lip depressor (AU116)**

Full model: full.AU116=glmmTMB(AU116_PropDurFv ~ Species*Relationship strength + Age.z +

Sex + Session + (1|AnimalID)+ (1|PersonID)+ (1|Dyad), family=beta_family, data=Fvdata)

Full-null model comparison: χ²=3.25, Df=3, *p=*0.35

Reduced-null model comparison: χ²=2.19, Df=2, *p=*0.33

### **Lip pucker (AU118)**

Full model: full.AU118.binary <- glmmTMB(AU118_BINARY ~ Species*Bonding + Age.z + Sex +
 Session + (1|AnimalID)+(1+ Age.z + Sex.W||PersonID)+ (1|Dyad),

family=binomial, data=Fvdata)

Full-null model comparison: χ²=2.78, Df=3, *p=*0.43

Reduced-null model comparison: χ²=2.33, Df=2, *p=*0.31

### **Lip corner puller (AU12)**

Full model: full.EAD101=glmmTMB(EAD101_PropDurFv ~ Species*Relationship strength + Age.z +

Sex + Session + (1|AnimalID)+ (1+Age.z + Sex.W||PersonID)+ (1|Dyad),

family=beta_family, data=Fvdata)

Full-null model comparison: χ²=9.01, Df=3, *p=*0.29

Reduced-null model comparison: χ²=1.53, Df=2, *p=*0.46

### **Panting (AD126)**

Full model: full.AD126=glmmTMB(AD126_PropDurFv ~ Species*Relationship strength + Age.z +

Sex + Session + (1|AnimalID)+ (1+ Age.z + Sex.W||PersonID)+ (1|Dyad), family=beta_family, data=Fvdata)

Full-null model comparison: χ²=0, Df=0, *p=*1

Reduced-null model comparison: χ²=0.11, Df=1, *p=*0.74

### **Blink (AU145)**

Full model: full.AU145_all=glmmTMB(AU145_All_SumFreq ~ Species*Relationship strength +

Age.z + Sex + Session + offset(FaceVisible_Dur.z)+ (1|AnimalID)+ (1+ Age.z +

Sex.W||PersonID) + (1|Dyad),family=nbinom1, data=Fvdata)

Full-null model comparison: χ²=0.16, Df=3, *p=*0.98

Reduced-null model comparison: χ²=0.04, Df=2, *p=*0.98

### **Tongue show (AD19)**

Full model: full.AD19=glmmTMB(AD19_PropDurFv ~ Species*Relationship strength + Age.z + Sex + Session +

(1|AnimalID)+ (1+ Age.z + Sex.W||PersonID)+ (1|Dyad), family=beta_family,
 data=Fvdata)

Full-null model comparison: χ²=2.56, Df=3, *p=*0.41

Reduced-null model comparison: χ²=2.81, Df=2, *p=*0.25

### **Lip wipe (AD37)**

Full model: full.zi.AD37_both <- glmmTMB(AD37_both_Freq ~ Session*Relationship strength +

Age.z + Sex + Session +offset(FaceVisible_Dur.z)+ (1|AnimalID)+ (1|PersonID)+

(1|Dyad), ziformula= ~ Species, data = Fvdata, family = poisson)

Full-null model comparison: χ²=2.59, Df=2, *p=*0.27

Reduced-null model comparison: χ²=2.66, Df=2, *p=*0.27

### **Nose wrinkle and upper lip raiser (AU109 + AU110)**

Full model: full.AU109_110.binary <- glmmTMB(AU109.AU110_BINARY ~ Species*Relationship

strength + Age.z + Sex + Session + (1|AnimalID)+ (1|PersonID)+ (1|Dyad),

family=binomial, data=Fvdata)

Full-null model comparison: χ²=1.59, Df=3, *p=*0.66

Reduced-null model comparison: χ²=0.02, Df=2, *p=*0.99

### **Yawn / Mouth stretch (AU27)**

Full model: full.zi.AU27 <- glmmTMB(AU27_Freq ~ Species*Relationship strength + Age.z + Sex +

Session +offset(FaceVisible_Dur.z)+ (1|AnimalID)+ (1+Age.z + Sex.W||PersonID)+

(1|Dyad), ziformula= ~ Species, data = Fvdata, family = poisson)

Full-null model comparison: χ²=8.75, Df=2, *p=*0.013

Full model summary:

| Variable | Estimate | Std. Error | z-value | p-value |
| --- | --- | --- | --- | --- |
| SpeciesWolf:Relationship strengthFH | -25.641 | 1.644e+05 | 0 | 0.9999 |

Reduced-null model comparison: χ²=12.05, Df=2, *p=*0.0024

Reduced model summary:

|  | Estimate | Std. Error | z-value | p-value |  | Lower CI | Upper CI |
| --- | --- | --- | --- | --- | --- | --- | --- |
| (Intercept) | -1.103 | 0.547 | -2.017 | 0.0437 | * | -2.915 | 0.142 |
| SpeciesWolf | 1.133 | 0.619 | 1.831 | 0.107 |  | -1.365 | 2.922 |
| **Relationship strength**FH | -1.810 | 0.768 | -2.358 | 0.018 | * | -3.954 | -0.380 |
| Age.z | 0.190 | 0.315 | 0.603 | 0.547 |  | -0.642 | 0.969 |
| SexW | 0.970 | 1.060 | 0.915 | 0.357 |  | -1.056 | 3.230 |
| Sessiontrial2 | 0.031 | 0.357 | 0.087 | 0.931 |  | -0.681 | 0.678 |

Pairwise comparison:

| contrast | estimate | SE | df | z.ratio | p-value |
| --- | --- | --- | --- | --- | --- |
| Relationship strength – Familiar | 1.81 | 0.768 | Inf | 2.358 | 0.0184 |

### **Nose licks (AD137)**

Full model: full.AD137=glmmTMB(AD137_Freq ~ Species*Relationship strength + Age.z + Sex +

Session + offset(FaceVisible_Dur.z)+ (1|AnimalID)+ (1+Sex.W + Age.z ||PersonID)+

(1|Dyad), family=nbinom1, data=Fvdata)

Full-null model comparison: χ²=6.15, Df=3, *p=*0.10

Reduced-null model comparison: χ²=7.35, Df=2, *p=*0.025

Reduced model summary:

|  | Estimate | Std. Error | z-value | p-value |  | Lower CI | Upper CI |
| --- | --- | --- | --- | --- | --- | --- | --- |
| (Intercept) | 0.786 | 0.403 | 1.950 | 0.051 | . | -0.161 | 1.533 |
| SpeciesWolf | -0.419 | 0.632 | -0.663 | 0.507 |  | -1.749 | 0.976 |
| **Relationship strength**FH | -0.630 | 0.272 | -2.317 | 0.021 | * | -1.251 | -0.107 |
| Age.z | -0.268 | 0.296 | -0.907 | 0.364 |  | -0.912 | 0.366 |
| SexW | -0.124 | 0.432 | -0.286 | 0.775 |  | -1.097 | 0.739 |
| Sessiontrial2 | -0.437 | 0.278 | -1.572 | 0.116 |  | -0.948 | 0.012 |

Pairwise comparison:

| contrast | estimate | SE | df | z.ratio | p-value |
| --- | --- | --- | --- | --- | --- |
| Relationship strength – FH | 0.63 | 0.272 | Inf | 2.317 | 0.0205 |

### **Ears forward (EAD101)**

Full model: full.AU110.binary <- glmmTMB(AU110_BINARY ~ Species*Relationship strength +

Age.z + Sex + Session + (1|AnimalID)+ (1+ Age.z + Sex.W||PersonID)+ (1|Dyad), family=binomial, data=Fvdata)

Full-null model comparison: χ²=8.94, Df=3, *p=*0.03

Full model summary:

| Variable | Estimate | Std. Error | z-Value | *P-value* |
| --- | --- | --- | --- | --- |
| SpeciesWolf:Relationship strengthFH | 0.1370 | 0.4616 | 0.297 | 0.7666 |

Reduced-null model comparison: χ²=8.84, Df=2, *p=*0.012

Reduced model summary:

|  | Estimate | Std. Error | z-value | p-value |  | Lower CI | Upper CI |
| --- | --- | --- | --- | --- | --- | --- | --- |
| (Intercept) | -1.606 | 0.316 | -5.086 | <0.001 | *** | -2.267 | -1.096 |
| **Species**Wolf | 0.752 | 0.340 | 2.209 | 0.027 | * | 0.082 | 1.407 |
| **Relationship strength**FH | 0.544 | 0.233 | 2.334 | 0.020 | * | 0.084 | 1.034 |
| Age.z | 0.083 | 0.169 | 0.491 | 0.624 |  | -0.240 | 0.426 |
| SexW | 0.252 | 0.249 | 1.014 | 0.311 |  | -0.251 | 0.797 |
| Sessiontrial2 | 0.389 | 0.223 | 1.746 | 0.081 | . | -0.057 | 0.836 |

Pairwise comparison:

| contrast | estimate | SE | df | z.ratio | p-value |
| --- | --- | --- | --- | --- | --- |
| Dog – Wolf | -0.834 | 0.363 | Inf | -2.297 | 0.0216 |
| Bonded – Familiar | -0.561 | 0.249 | Inf | -2.250 | 0.0245 |

### **Ears Adductor (EAD102)**

Full model: full.EAD102=glmmTMB(EAD102_PropDurFv ~ Species*Relationship strength + Age.z +

Sex + Session + (1|AnimalID)+ (1|PersonID)+ (1|Dyad), family=beta_family, data=Fvdata)

Full-null model comparison: χ²=4.33, Df=3, *p=*0.23

Reduced-null model comparison: χ²=0.46, Df=2, *p=*0.79

### **Ears Flattener (EAD103)**

Full model: full.EAD103=glmmTMB(EAD103_PropDurFv ~ Species*Relationship strength + Age.z +

Sex + Session + (1|AnimalID)+c(1+ Age.z + Sex.W||PersonID)+ (1|Dyad),

family=beta_family, data=Fvdata)

Full-null model comparison: χ²=7.40, Df=3, *p=*0.06

Full model summary:

| Variable | Estimate | Std. Error | z-value | p-value |
| --- | --- | --- | --- | --- |
| SpeciesWolf:Relationship strengthFH | 0.66422 | 0.46891 | 1.417 | 0.1566 |

Reduced-null model comparison: χ²=3.91, Df=2, *p=*0.048

Reduced model summary:

|  | Estimate | Std. Error | z-value | p-value |  | Lower CI | Upper CI |
| --- | --- | --- | --- | --- | --- | --- | --- |
| (Intercept) | -1.540 | 0.288 | -5.355 | <0.001 | *** | -2.218 | -0.920 |
| SpeciesWolf | -0.471 | 0.317 | -1.486 | 0.137 |  | -1.172 | 0.186 |
| **Relationship strength**FH | -0.489 | 0.235 | -2.082 | 0.037 | * | -0.984 | -0.038 |
| Age.z | -0.035 | 0.158 | -0.219 | 0.827 |  | -0.366 | 0.320 |
| SexW | -0.241 | 0.247 | -0.975 | 0.330 |  | -0.719 | 0.257 |
| Sessiontrial2 | 0.072 | 0.229 | 0.316 | 0.752 |  | -0.414 | 0.570 |

Pairwise comparison:

| contrast | estimate | SE | df | z.ratio | p-value |
| --- | --- | --- | --- | --- | --- |
| Bonded - Familiar | 0.489 | 0.235 | Inf | 2.082 | 0.037 |

### **Ears Rotator (EAD104)**

Full model: full.EAD104_All=glmmTMB(EAD104_All_PropSumDurFv ~ Species*Relationship

strength + Age.z + Sex + Session + (1|AnimalID)+ (1+ Age.z + Sex.W||PersonID)+

(1|Dyad), family=beta_family, data=Fvdata)

Full-null model comparison: χ²=7.72, Df=3, *p=*0.052

Full model summary:

| Variable | Estimate | Std. Error | z-value | p-value |
| --- | --- | --- | --- | --- |
| SpeciesWolf:Relationship strengthFH | -0.11904 | 0.49919 | -0.238 | 0.811513 |

Reduced-null model comparison: χ²=7.66, Df=2, *p=*0.022

Reduced model summary:

|  | Estimate | Std. Error | z-value | p-value |  | Lower CI | Upper CI |
| --- | --- | --- | --- | --- | --- | --- | --- |
| (Intercept) | -0.060 | 0.313 | -0.191 | 0.848 |  | -0.661 | 0.556 |
| **Species**Wolf | -1.251 | 0.376 | -3.326 | 0.001 | *** | -2.054 | -0.514 |
| Relationship strengthFH | 0.049 | 0.220 | 0.221 | 0.825 |  | -0.425 | 0.486 |
| **Age.z** | 0.670 | 0.183 | 3.663 | 0.000 | *** | 0.285 | 1.067 |
| SexW | 0.070 | 0.341 | 0.205 | 0.837 |  | -0.666 | 0.692 |
| Sessiontrial2 | 0.209 | 0.218 | 0.960 | 0.337 |  | -0.216 | 0.618 |

Pairwise comparison:

| contrast | estimate | SE | df | z.ratio | p-value |
| --- | --- | --- | --- | --- | --- |
| Dog – Wolf | 1.25 | 0.376 | Inf | 3.326 | 0.0009 |

### **Ears Downward (EAD105)**

Full model: full.EAD105=glmmTMB(EAD105_PropDurFv ~ Species*Relationship strength + Age.z +

Sex + Session + (1|AnimalID)+ (1 + Age.z + Sex.W||PersonID)+ (1|Dyad),

family=beta_family, data=Fvdata)

Full-null model comparison: χ²=7.01, Df=3, *p=*0.07

Full model summary:

| Variable | Estimate | Std. Error | z-value | p-value |
| --- | --- | --- | --- | --- |
| SpeciesWolf:Relationship strengthFH | -0.43184 | 0.53909 | -0.801 | 0.4231 |

Reduced-null model comparison: χ²=6.37, Df=2, *p=*0.04

Reduced model summary:

|  | Estimate | Std. Error | z-value | p-value |  | Lower CI | Upper CI |
| --- | --- | --- | --- | --- | --- | --- | --- |
| (Intercept) | -1.436 | 0.256 | -5.620 | 0.000 | *** | -1.992 | -0.962 |
| **Species**Wolf | -0.852 | 0.304 | -2.807 | 0.005 | ** | -1.516 | -0.267 |
| Relationship strengthFH | -0.148 | 0.247 | -0.599 | 0.549 |  | -0.651 | 0.309 |
| Age.z | 0.351 | 0.197 | 1.782 | 0.075 | . | -0.025 | 0.719 |
| SexW | -0.179 | 0.212 | -0.843 | 0.399 |  | -0.619 | 0.249 |
| Sessiontrial2 | -0.400 | 0.210 | -1.900 | 0.058 | . | -0.869 | -0.018 |

Pairwise comparison:

| contrast | estimate | SE | df | z.ratio | p-value |
| --- | --- | --- | --- | --- | --- |
| Dog – Wolf | 0.927 | 0.31 | Inf | 2.994 | 0.0028 |

## Statistical analysis 3.2: CF-WSC Dogs – Pet Dog comparison

Here, we only compared the facial expressions that had differed between the CF-WSC Dogs and Wolves to check for the effect of morphological differences (Floppy ears in the CF-WSC dogs vs. Upright ears in the Pet dogs (and CF-WSC wolves)

### **Inner brow raiser (AU101)**

Full model: full.AU101=glmmTMB(AU101_PropDurFvv ~ Species*Relationship strength + Age.z +

Sex + Session + (1|AnimalID)+ (1|PersonID)+ (1|Dyad), family=beta_family,

data=Fvdata)

Full-null model comparison: χ²=5.21, Df=3, *p=*015

Reduced-null model comparison: χ²=4.64, Df=2, *p=*0.098

Reduced model summary:

|  | Estimate | Std. Error | z-value | p-value |  | Lower CI | Upper CI |
| --- | --- | --- | --- | --- | --- | --- | --- |
| SpeciesWSC_Dogs | -0.095 | 0.310 | -0.307 | 0.759 |  | -0.741 | 0.529 |

### **Upper lip raiser (AU102)**

Full model: full.AU110.binary <- glmmTMB(AU110_BINARY ~ Species*Relationship strength +

Age.z + Sex + Session + (1|AnimalID)+ (1|PersonID)+ (1|Dyad), family=binomial, data=Fvdata)

Full-null model comparison: χ²=4.77, Df=3, *p=*0.19

Reduced-null model comparison: χ²=4.77, Df=2, *p=*0.09

Reduced model summary:

|  | Estimate | Std. Error | z-value | p-value |  | Lower CI | Upper CI |
| --- | --- | --- | --- | --- | --- | --- | --- |
| SpeciesWSC_Dogs | 0.998 | 0.668 | 1.495 | 0.135 |  | -0.205 | 2.912 |

### **Ears forward (EAD101)**

Full model: full.EAD101=glmmTMB(EAD101_PropDurFv ~ Species*Relationship strength + Age.z +

Sex + Session + (1|AnimalID)+ (1|PersonID)+ (1|Dyad), family=beta_family,

data=Fvdata)

Full-null model comparison: χ²=6.14, Df=3, *p=*0.11

Reduced-null model comparison: χ²=6.04, Df=2, *p=*0.049

Reduced model summary:

|  | Estimate | Std. Error | z-value | p-value |  | Lower CI | Upper CI |
| --- | --- | --- | --- | --- | --- | --- | --- |
| SpeciesWSC_Dogs | -0.668 | 0.4100 | -1.628 | 0.1034 |  | -1.420 | 0.168 |

Pairwise comparison:

|  | Estimate | Std. Error | df | z-ratio | p-value |
| --- | --- | --- | --- | --- | --- |
| Pet – WSC dog | 0.668 | 0.41 | Inf | 1.628 | 0.1034 |

### **Ear Rotator (EAD104)**

Full model: full.EAD104_All=glmmTMB(EAD104_All_PropSumDurFv ~ Species*Relationship

strength + Age.z + Sex + Session + (1|AnimalID)+ (1|PersonID)+ (1|Dyad),

family=beta_family, data=Fvdata)

Full-null model comparison: χ²=1.38, Df=3, *p=*0.71

Reduced-null model comparison: χ²=1.37, Df=2, *p=*0.50

Reduced model summary:

|  | Estimate | Std. Error | z-value | p-value |  | Lower CI | Upper CI |
| --- | --- | --- | --- | --- | --- | --- | --- |
| SpeciesWSC_Dogs | 0.37939 | 0.32757 | 1.158 | 0.2468 |  | -0.250 | 1.097 |

### **Ears downward (EAD105)**

Full model: full.EAD105=glmmTMB(EAD105_PropDurFv ~ Species*Relationship strength + Age.z +

Sex + Session + (1|AnimalID)+ (1|PersonID)+ (1|Dyad), family=beta_family,

data=Fvdata)

Full-null model comparison: χ²=2.32, Df=3, *p=*0.51

Reduced-null model comparison: χ²=2.32, Df=2, *p=*0.32

|  | Estimate | Std. Error | z-value | p-value |  | Lower CI | Upper CI |
| --- | --- | --- | --- | --- | --- | --- | --- |
| SpeciesWSC_Dogs | 0.38386 | 0.25666 | 1.496 | 0.135 |  | -0.112 | 0.954 |

## Statistical analysis 3.3: Human facial reactions towards dogs vs. wolves

### **Expressiveness: Facial change (sum of all AU frequencies)**

Full model: full.ExpFreq=glmmTMB(Expressions_SumFreq ~ Relationship strength*Species +

Age.z + Session + offset(Animal_Prox_both_dur.z)+ (1|AnimalID)+ (1|PersonID,

family=nbinom2, data=xdata)

Full-null model comparison: χ²=10.6, Df=3, *p=*0.014

Full model summary:

| Variable | Estimate | Std. Error | z-value | p-value |
| --- | --- | --- | --- | --- |
| SpeciesWolf:Relationship strengthFH | -0.404 | 0.303 | -1.316 | 0.1882 |

Reduced-null model comparison: χ²=8.89, Df=2, *p=*0.012

Reduced model summary:

|  | Estimate | Std. Error | z-value | p-value |  | Lower CI | Upper CI |
| --- | --- | --- | --- | --- | --- | --- | --- |
| (Intercept) | 4.276 | 0.273 | 15.657 | 0.000 | *** | 3.721 | 4.781 |
| **Species**Wolf | 0.449 | 0.158 | 2.846 | 0.004 | ** | 0.154 | 0.753 |
| Relationship strengthFH | 0.245 | 0.388 | 0.633 | 0.527 |  | -0.556 | 0.970 |
| Age.z | 0.262 | 0.112 | 2.344 | 0.019 | * | 0.003 | 0.488 |
| Sessiontrial2 | -0.399 | 0.136 | -2.935 | 0.003 | ** | -0.688 | -0.148 |

Pairwise comparison:

| contrast | estimate | SE | df | z.ratio | p-value |
| --- | --- | --- | --- | --- | --- |
| Dog - Wolf | 0.449 | 0.158 | Inf | -2.846 | 0.0044 |

### **Expressiveness: Facial range (sum of all occurred AUs)**

Full model: full.ExpFreqBB=glmer(Expressivity_SumFreqBINARY ~ Relationship strength

+ Species + Age.z + Session + offset(Animal_Prox_both_dur.z)+ (1|AnimalID)+ (1|PersonID)+ (1|Dyad), family=nbinom2, data=xdata)

Full-null model comparison: χ²=18.0, Df=3, *p=*0.00044

Full model summary:

| Variable | Estimate | Std. Error | z-value | p-value |
| --- | --- | --- | --- | --- |
| **Species**Wolf:**Relationship strength**FH | -0.7803 | 0.4089 | -1.904 | 0.0569 |

Pairwise comparison

| contrast | estimate | SE | df | z.ratio | p-value |
| --- | --- | --- | --- | --- | --- |
| **Dog Bonded - Wolf Bonded** | -1.320 | 0.223 | Inf | -5.928 | <.0001 |
| Dog Bonded - Dog Familiar | -0.520 | 0.591 | Inf | -0.881 | 0.8148 |
| Dog Bonded - Wolf Familiar | -1.060 | 0.585 | Inf | -1.81 | 0.2683 |
| Wolf Bonded - Dog Familiar | 0.799 | 0.603 | Inf | 1.324 | 0.5474 |
| Wolf Bonded - Wolf Familiar | 0.260 | 0.594 | Inf | 0.438 | 0.972 |
| Dog Familiar - Wolf Familiar | -0.539 | 0.351 | Inf | -1.536 | 0.4159 |

Reduced-null model comparison: χ²=14.376, Df=2, *p=*0.00076

Reduced model summary:

|  | Estimate | Std. Error | z-value | p-value |  | Lower CI | Upper CI |
| --- | --- | --- | --- | --- | --- | --- | --- |
| (Intercept) | 2.404 | 0.431 | 5.579 | <0.001 | *** | 1.514 | 3.257 |
| SpeciesWolf | 1.104 | 0.193 | 5.724 | <0.001 | *** | 0.709 | 1.520 |
| Relationship strengthFH | 0.138 | 0.613 | 0.226 | 0.821 |  | -1.092 | 1.406 |
| Age.z | 0.311 | 0.140 | 2.225 | 0.026 | * | -0.010 | 0.589 |
| Sessiontrial2 | -0.528 | 0.169 | -3.126 | 0.002 | ** | -0.865 | -0.198 |

Pairwise comparison:

| contrast | estimate | SE | df | z.ratio | p-value |
| --- | --- | --- | --- | --- | --- |
| Dog - Wolf | -1.1 | 0.193 | Inf | -5.724 | <.0001 |

### **Intensity Ratio**

Full model: full.IntRatFreq=glmmTMB(Ratio_HLIntensity_Freq_log_beta ~ Species*Relationship

strength + Session + Age.z + (1|AnimalID)+ (1|PersonID)+ (1|Dyad), family=beta_family, data=xdata)

Full-null model comparison: χ²=7.43, Df=3, *p=*0.06

Full model summary:

| Variable | Estimate | Std. Error | z-value | p-value |
| --- | --- | --- | --- | --- |
| SpeciesWolf:Relationship strengthFH | -0.1520 | 0.371224 | -0.410 | 0.682 |

Reduced-null model comparison: χ²=7.26, Df=2, *p=*0.027

Reduced model summary:

|  | Estimate | Std. Error | z-value | p-value | value | Lower CI | Upper CI |
| --- | --- | --- | --- | --- | --- | --- | --- |
| (Intercept) | -2.125 | 0.213 | -9.970 | 0.000 | *** | -2.577 | -1.717 |
| Relationship strengthFH | -0.066 | 0.314 | -0.210 | 0.834 |  | -0.717 | 0.545 |
| **Species**Wolf | -0.648 | 0.217 | -2.991 | 0.003 | ** | -1.099 | -0.235 |
| Age.z | 0.026 | 0.146 | 0.179 | 0.858 |  | -0.319 | 0.293 |
| Sessiontrial2 | 0.039 | 0.163 | 0.236 | 0.813 |  | -0.270 | 0.376 |

Pairwise comparison:

| contrast | estimate | SE | df | z.ratio | p-value |
| --- | --- | --- | --- | --- | --- |
| Dog - Wolf | 0.648 | 0.217 | Inf | 2.991 | 0.0028 |

### **Valence: Duration of AUs associated with negative emotions (Ekman et al., 2002)**

Full model: full.PNegativeDur=glmmTMB(Negative_PropDurV_beta ~ Species*Relationship

strength + Session + Age.z + (1|AnimalID)+ (1|PersonID)+ (1|Dyad),

family=beta_family, data=xdata)

Full-null model comparison: χ²=0.88, Df=3, *p=*0.83

Reduced-null model comparison: χ²=0.29, Df=2, *p=*0.87

Reduced model summary:

| Variable | Estimate | Std. Error | z-value | p-value |
| --- | --- | --- | --- | --- |
| SpeciesWolf | 0.02329 | 0.20146 |  |  |

### **Valence: Duration of AUs associated with positive emotions (Ekman et al., 2002)**

Full model: full.PPositiveDur=glmmTMB(PurePositive_PropDurV_beta ~ Species*Relationship

strength + Session + Age.z + (1|AnimalID)+ (1PersonID)+ (1|Dyad),

family=beta_family, data=xdata)

Full-null model comparison: χ²=9.54, Df=3, *p=*0.023

Full model summary:

|  | Estimate | Std. Error | z-value | p-value |  | Lower CI | Upper CI |
| --- | --- | --- | --- | --- | --- | --- | --- |
| **Species**Wolf**:Relationship**  **strength**FH | -0.865 | 0.473 | -1.828 | 0.068 | . | -1.852 | 0.026 |

Pairwise comparison:

| contrast | estimate | SE | df | z.ratio | p-value |
| --- | --- | --- | --- | --- | --- |
| Dog Bonded - Wolf Bonded | 0.445 | 0.393 | Inf | 1.132 | 0.6697 |
| **Dog Bonded - Dog Familiar** | -1.115 | 0.366 | Inf | -3.051 | 0.0122 |
| Dog Bonded - Wolf Familiar | 0.194 | 0.433 | Inf | 0.449 | 0.9698 |
| Wolf Bonded - Dog Familiar | -1.56 | 0.499 | Inf | -3.129 | 0.0095 |
| Wolf Bonded - Wolf Familiar | -0.251 | 0.387 | Inf | -0.648 | 0.9162 |
| **Dog Familiar - Wolf Familiar** | 1.31 | 0.471 | Inf | 2.781 | 0.0278 |

Reduced model summary:

|  | Estimate | Std. Error | z-value | p-value | value | Lower CI | Upper CI |
| --- | --- | --- | --- | --- | --- | --- | --- |
| (Intercept) | -0.648 | 0.299 | -2.164 | 0.030 |  | -1.244 | -0.020 |
| Relationship strengthFH | 0.735 | 0.444 | 1.656 | 0.097 |  | -0.099 | 1.665 |
| **Species**Wolf | -0.751 | 0.344 | -2.182 | 0.029 | * | -1.460 | -0.121 |
| Age.z | 0.089 | 0.239 | 0.374 | 0.708 |  | -0.351 | 0.572 |
| Sessiontrial2 | -0.598 | 0.222 | -2.687 | 0.007 | ** | -1.034 | -0.150 |

Pairwise comparison:

| contrast | estimate | SE | df | z.ratio | p-value |
| --- | --- | --- | --- | --- | --- |
| Dog - Wolf | 0.752 | 0.345 | Inf | 2.182 | 0.0291 |
| Trial1 – Trial2 | 0.599 | 0.223 | Inf | 2.687 | 0.0072 |

# **References**

Dobson, A. J., & Barnett, A. G. (2018). *An introduction to generalized linear models*. Chapman and Hall/CRC.

Ekman, P., Friesen, W., & Hager, J. (2002). *Facial Action Coding System - The Manual*.

Lenth, R. (2023). emmeans: Estimated Marginal Means, aka Least-Squares Means. R package, version 1.9.0. <https://CRAN.R-project.org/package=emmeans>

Pedretti, G., Canori, C., Costantini, E., Palme, R., Valsecchi, P., & Marshall-Pescini, S. (2024). Intra and interspecific audience effect on domestic dogs' behavioural displays and facial expressions. *Scientific Reports*, *14*(1), 9546.

Waller, B. M., Peirce, K., Caeiro, C. C., Scheider, L., Burrows, A. M., McCune, S., & Kaminski, J. (2013). Paedomorphic facial expressions give dogs a selective advantage. *PLOS ONE*, *8*(12), e82686.
